# Supplementary material for: Rapid Detection of Avocado Oil Adulteration Using Low-Field Nuclear Magnetic Resonance
Source: Foods. 2022 Apr 14;11(8):1134. doi: 10.3390/foods11081134 (PMC9032617; doi:10.3390/foods11081134)
Supplement: Supplementary file 1 [file foods-11-01134-s001.zip › foods-1623522-supplementary.pdf]

Article

Rapid detection of avocado oil adulteration using low-field nuclear magnetic resonance

Table S1. The preparation of oil samples.

Oil samples	Adulteration levels/%	Calibration Members	Validation Members
Pure AO (4 brands)	0	15	5
Pure SO	100	4	1
Pure CO	100	4	1
Pure RO	100	4	1
AO-SO	10	7	3
	20	7	3
	30	7	3
	40	7	3
	60	7	3
	80	7	3
AO-CO	10	7	3
	20	7	3
	30	7	3
	40	7	3
	60	7	3
	80	7	3
AO-RO	10	7	3
	20	7	3
	30	7	3
	40	7	3
	60	7	3
	80	7	3

Note: The AO used in adulterated samples (AO-SO, AO-CO and AO-RO) was randomly selected from four brands of AO.

Table S2. Changes of LF-NMR parameters of AO at different adulteration levels with SO, CO or RO.

Oil samples	Adulteration level/%	T_{2W}/ms	T_{21P}/ms	T_{22P}/ms	T_{23P}/ms	P_{21}/%	P_{22}/%	P_{23}/%
AO-SO	10	116.63 ± 4.41^{f}	2.20 ± 0.11^{cd}	65.03 ± 1.33^{d}	209.59 ± 5.64^{f}	2.24 ± 0.16^{bc}	61.12 ± 1.33^{a}	36.64 ± 1.18^{f}
	20	121.10 ± 3.74^{e}	2.13 ± 0.06^{d}	65.57 ± 1.61^{cd}	216.51 ± 3.95^{e}	2.18 ± 0.12^{c}	60.13 ± 1.09^{ab}	37.69 ± 0.98^{def}
	30	122.89 ± 3.36^{de}	2.19 ± 0.05^{cd}	66.28 ± 1.54^{cd}	218.88 ± 3.76^{de}	2.32 ± 0.12^{ab}	59.62 ± 1.03^{bc}	38.06 ± 0.92^{de}
	40	126.22 ± 3.98^{dc}	2.19 ± 0.07^{cd}	66.83 ± 1.64^{cd}	223.72 ± 4.96^{d}	2.34 ± 0.09^{ab}	58.85 ± 0.99^{cd}	38.81 ± 0.91^{cd}
	60	130.36 ± 3.08^{c}	2.23 ± 0.03^{c}	67.55 ± 1.16^{c}	229.87 ± 4.28^{c}	2.40 ± 0.06^{a}	57.95 ± 0.70^{d}	39.65 ± 0.65^{c}
	80	137.62 ± 3.86^{b}	2.18 ± 0.05^{cd}	69.42 ± 1.70^{b}	238.80 ± 4.45^{b}	2.40 ± 0.08^{a}	56.39 ± 0.82^{e}	41.21 ± 0.75^{b}
	100	147.92 ± 4.88^{a}	3.26 ± 0.05^{a}	72.71 ± 1.94^{a}	251.98 ± 4.58^{a}	1.85 ± 0.06^{d}	55.49 ± 1.08^{e}	42.66 ± 1.05^{a}
AO-CO	10	119.34 ± 2.77^{e}	2.44 ± 0.17^{e}	65.92 ± 1.13^{c}	214.75 ± 3.69^{e}	2.14 ± 0.05^{b}	61.06 ± 0.70^{a}	36.80 ± 0.66^{e}
	20	120.46 ± 4.85^{e}	2.48 ± 0.10^{de}	66.29 ± 1.97^{c}	216.55 ± 5.66^{de}	2.17 ± 0.12^{b}	60.89 ± 1.14^{a}	36.94 ± 1.12^{e}
	30	123.16 ± 4.09^{de}	2.53 ± 0.16^{cde}	66.65 ± 1.76^{c}	221.28 ± 4.12^{d}	2.22 ± 0.13^{b}	60.33 ± 1.16^{ab}	37.45 ± 1.08^{de}
	40	127.75 ± 3.62^{cd}	2.51 ± 0.19^{de}	67.74 ± 1.61^{bc}	226.76 ± 4.09^{c}	2.18 ± 0.09^{b}	59.19 ± 0.81^{bc}	38.62 ± 0.78^{cd}
	60	129.89 ± 4.64^{c}	2.62 ± 0.17^{cd}	67.93 ± 1.94^{bc}	231.14 ± 4.89^{c}	2.23 ± 0.09^{ab}	58.93 ± 1.02^{c}	38.84 ± 1.01^{c}
	80	135.49 ± 4.79^{b}	2.68 ± 0.10^{6c}	69.61 ± 1.81^{ab}	236.22 ± 4.78^{b}	2.31 ± 0.07^{a}	57.23 ± 1.18^{d}	40.45 ± 1.16^{b}
	100	144.11 ± 5.44^{a}	3.37 ± 0.10^{a}	71.34 ± 1.90^{a}	243.72 ± 4.60^{a}	1.93 ± 0.05^{c}	55.11 ± 1.56^{e}	42.96 ± 1.52^{a}
AO-RO	10	120.42 ± 4.25^{a}	2.96 ± 0.09^{c}	67.01 ± 1.44^{b}	215.35 ± 4.77^{c}	1.88 ± 0.10^{bc}	61.32 ± 1.15^{a}	36.80 ± 1.13^{a}
	20	120.71 ± 4.85^{a}	2.93 ± 0.18^{c}	67.19 ± 1.73^{b}	215.94 ± 5.01^{c}	1.89 ± 0.09^{bc}	61.34 ± 1.41^{a}	36.77 ± 1.40^{a}
	30	120.38 ± 4.09^{a}	2.96 ± 0.14^{c}	66.82 ± 1.45^{b}	216.52 ± 4.60^{c}	1.90 ± 0.07^{bc}	61.54 ± 1.14^{a}	36.56 ± 1.11^{a}
	40	122.02 ± 4.41^{a}	3.01 ± 0.16^{bc}	67.74 ± 1.44^{ab}	217.70 ± 4.04^{bc}	1.92 ± 0.09^{b}	61.07 ± 1.43^{a}	37.01 ± 1.44^{a}
	60	123.28 ± 5.30^{a}	3.04 ± 0.10^{bc}	68.3 ± 1.64^{ab}	220.11 ± 5.62^{abc}	1.95 ± 0.05^{b}	61.02 ± 1.52^{a}	37.02 ± 1.53^{a}
	80	125.67 ± 5.40^{a}	3.13 ± 0.06^{b}	69.43 ± 1.83^{a}	223.10 ± 4.52^{ab}	2.08 ± 0.10^{a}	60.45 ± 1.70^{a}	37.47 ± 1.74^{a}
	100	125.17 ± 4.61^{a}	3.40 ± 0.03^{a}	69.33 ± 1.70^{a}	223.69 ± 3.48^{a}	1.82 ± 0.02^{c}	61.24 ± 1.51^{a}	36.94 ± 1.51^{a}

Note: Different lowercase letters (a-f) indicate a significant difference ($p < 0.05$). Data were presented as the mean ± standard error.

Figure S1. Comparison of key LF-NMR parameters of AO-SO, AO-CO and AO-RO in a heatmap made in GraphPad Prism 7. The largest value of each LF-NMR parameter was determined as 1, other values was shown as the specific value against the largest value. Different capital letters (A-F) indicate a significant difference ($p < 0.05$).

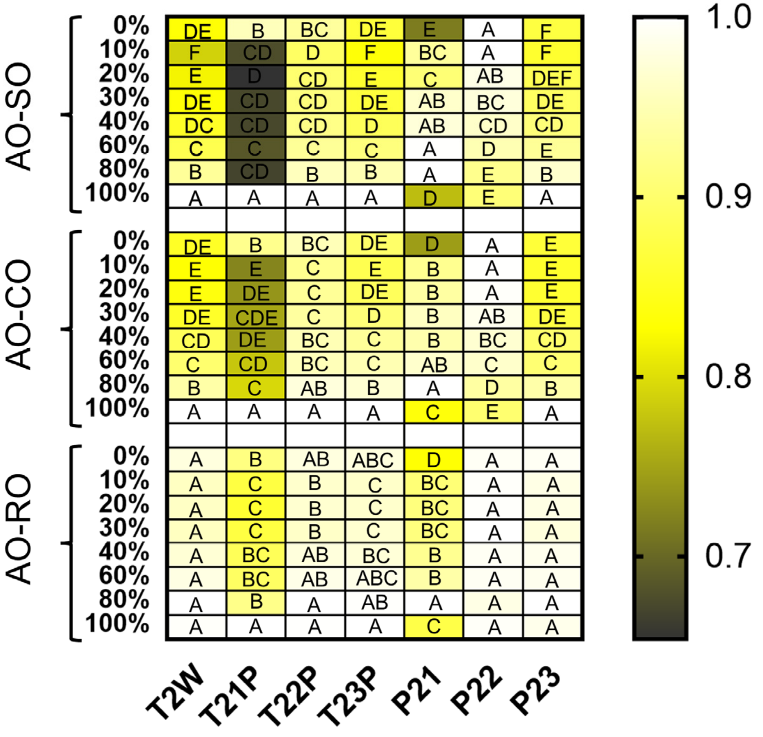

Figure S2. Detection of outliers in: a) AO, b) AO-SO, c) AO-CO and d) AO-RO populations making use of the Mahalanobis distance (DModX).

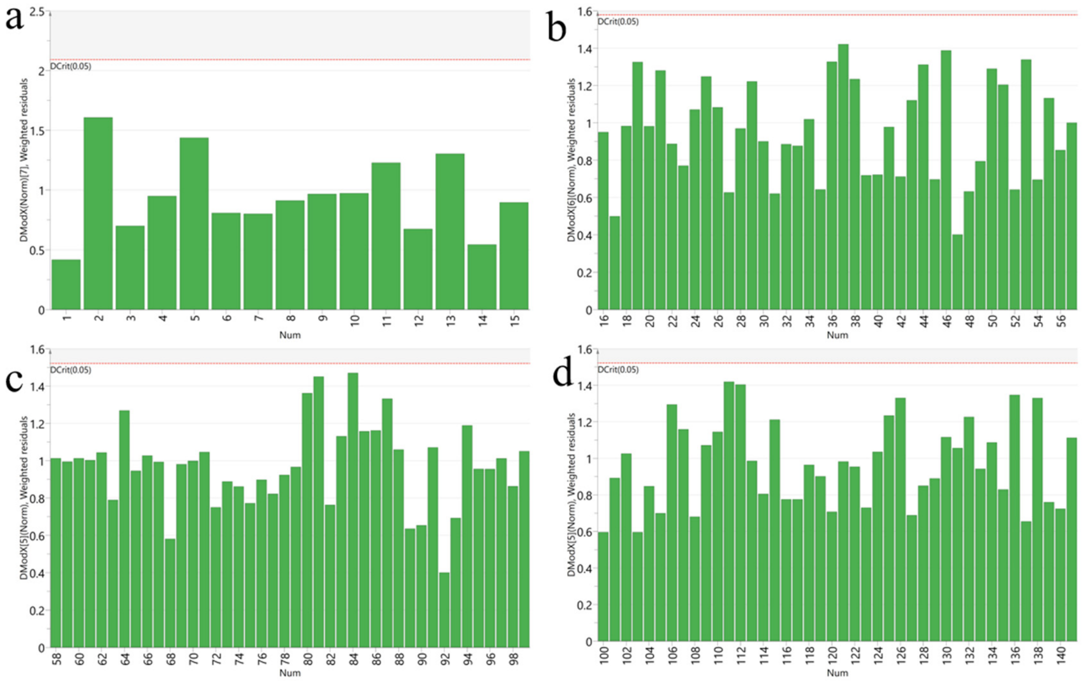

Figure S3. The ROC plot of AO, AO-SO, AO-CO and AO-RO in SIMCA analysis.

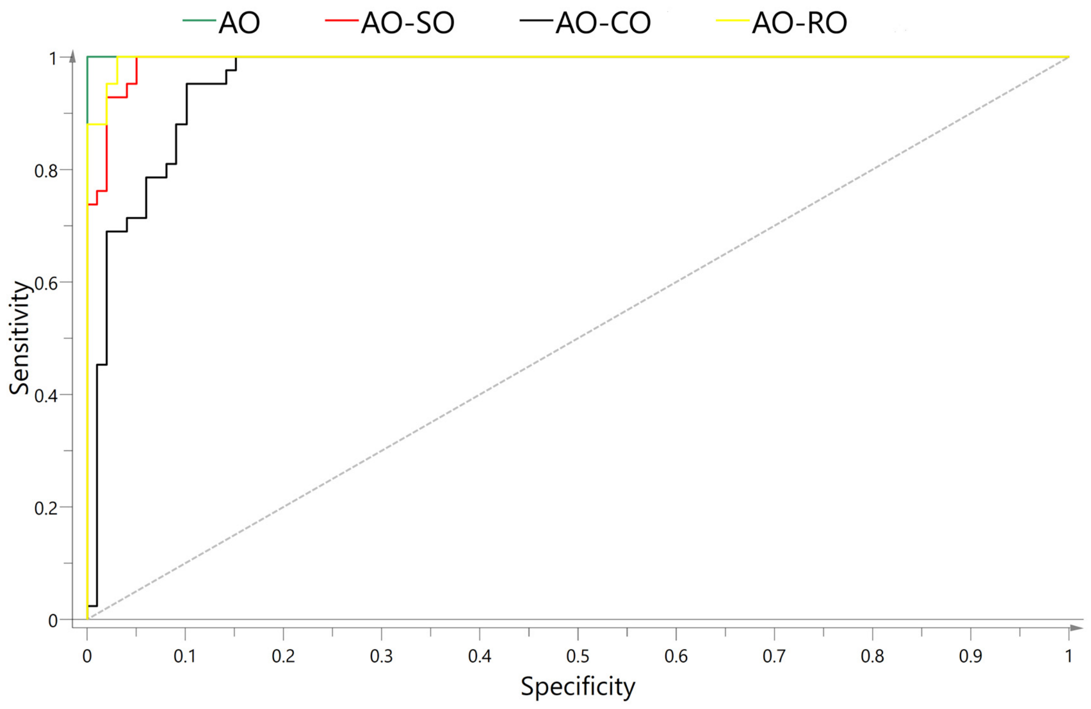

Figure S4. The standardized coefficients of AO-SO (a), AO-CO (b), AO-RO (c) and AO-SO/CO/RO (d) in PLSR analysis.

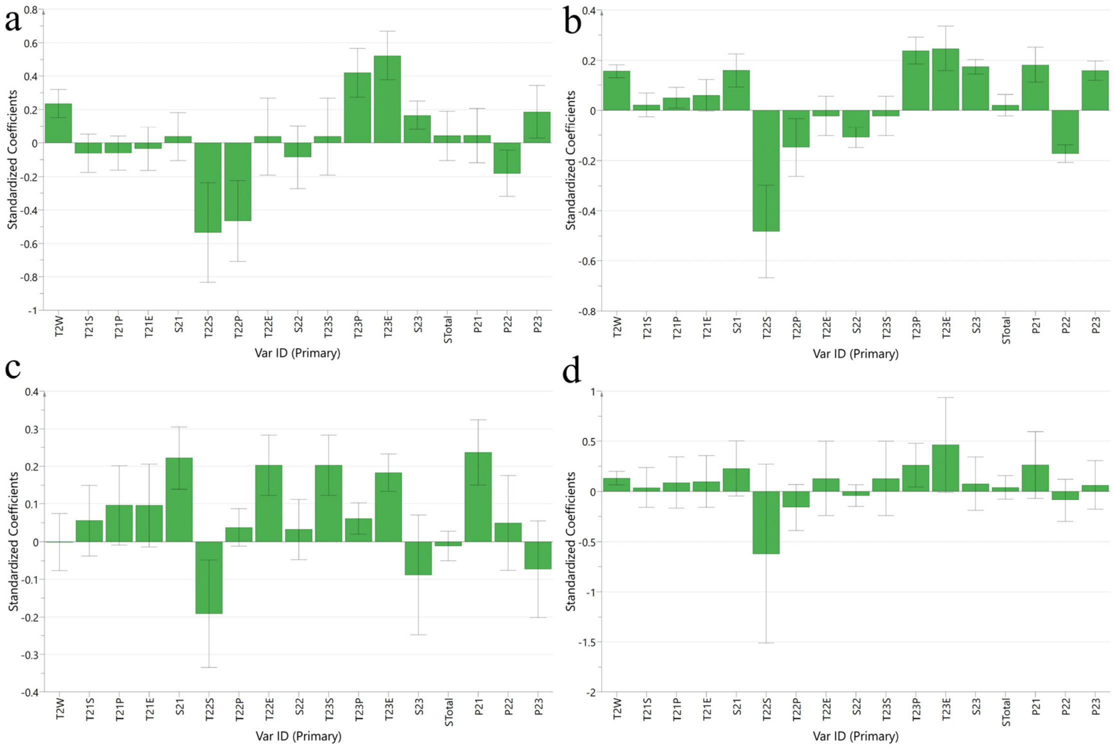

Table S3. Data used for the establishment of PCA model.

Number	Name	Group	T2W	T21S	T21P	T21E	S21	T22S	T22P	T22E	S22	T23S	T23P	T23E	S23	STotal	P21	P22	P23
1	AO-1	AO	115	2.8	3.14	3.45	141	41.8	64.8	133.7	5316.8	136.3	211.25	353.3	2905.5	8363.1	0.017	0.64	0.35
2	AO-2	AO	120	2.8	3.08	3.45	138	43.5	67.4	133.7	5135.7	136.3	215.31	360.1	3046.8	8320.6	0.017	0.62	0.37
3	AO-3	AO	126	2.69	2.96	3.32	146	45.1	68.7	136.3	5006.8	138.92	223.67	374.1	3140.8	8293.1	0.018	0.6	0.38
4	AO-4	AO	119	2.85	3.14	3.52	149	43.5	66.1	133.7	5102	136.3	215.31	360.1	3016.7	8267.3	0.018	0.62	0.37
5	ao-5	AO	124	2.85	3.14	3.52	148	45.1	68.7	136.3	4989.1	138.92	219.45	374.1	3097.2	8233.9	0.018	0.61	0.38
6	ao-6	AO	115	2.91	3.2	3.58	140	41.8	64.8	133.7	5271.1	136.3	211.25	353.3	2884.5	8295.9	0.017	0.64	0.35
7	ao-7	AO	126	2.8	3.08	3.45	145	45.1	68.7	136.3	4975.9	138.92	223.67	374.1	3112.4	8233.3	0.018	0.6	0.38
8	AO-8	AO	124	2.85	3.14	3.52	140	44.3	67.4	133.7	5015.3	136.3	219.45	367	3122.3	8277.5	0.017	0.61	0.38
9	AO-9	AO	128	2.75	3.08	3.38	142	46	70	136.3	4934	138.92	223.67	374.1	3178.6	8254.9	0.017	0.6	0.39
10	AO-10	AO	123	2.8	3.08	3.45	143	44.3	68.7	136.3	5063.5	138.92	219.45	367	3054.6	8261	0.017	0.61	0.37
11	AO-11	AO	128	2.75	3.08	3.38	146	46	70	136.3	4915	138.92	223.67	374.1	3173.6	8234.5	0.018	0.6	0.39
12	AO-12	AO	123	2.91	3.26	3.65	142	44.3	68.7	136.3	5037.1	138.92	219.45	367	3035.8	8214.7	0.017	0.61	0.37
13	AO-13	AO	118	2.85	3.14	3.52	133	42.6	66.1	133.7	5167.6	136.3	211.25	360.1	2999.8	8300.2	0.016	0.62	0.36
14	AO-14	AO	125	2.8	3.08	3.38	141	45.1	68.7	133.7	4998.2	136.3	219.45	360.1	3131	8269.9	0.017	0.6	0.38
15	AO-15	AO	129	2.8	3.08	3.45	144	46.9	70	136.3	4886.8	138.92	223.67	374.1	3210.7	8241.1	0.017	0.59	0.39
16	AO-10%SO-1	AO-SO	112	2.06	2.36	2.69	174	41	64.8	131.2	5488	133.73	203.35	346.6	3071.1	8733.3	0.02	0.63	0.35
17	AO-10%SO-2	AO-SO	115	2.06	2.36	2.69	183	41.8	64.8	131.2	5371.1	133.73	207.26	353.3	3143.9	8698.5	0.021	0.62	0.36
18	AO-10%SO-3	AO-SO	114	1.91	2.14	2.4	199	43.5	63.6	128.7	5440.7	131.21	207.26	340.1	3192.2	8832.3	0.023	0.62	0.36

19	AO-10%SO-4	AO-SO	116	1.91	2.18	2.45	200	43.5	64.8	128.7	5384	131.21	207.26	346.6	3225.6	8809.3	0.023	0.61	0.37
20	AO-10%SO-5	AO-SO	125	1.95	2.18	2.45	216	46.9	67.4	131.2	5152.9	133.73	219.45	360.1	3391	8760.2	0.025	0.59	0.39
21	AO-10%SO-6	AO-SO	114	1.91	2.1	2.36	195	43.5	63.6	128.7	5470.8	131.21	207.26	340.1	3190.7	8856.2	0.022	0.62	0.36
22	AO-10%SO-7	AO-SO	121	1.88	2.1	2.36	209	45.1	66.1	128.7	5296.3	131.21	215.31	346.6	3326.3	8831.8	0.024	0.6	0.38
23	AO-20%SO-1	AO-SO	115	1.95	2.18	2.45	175	42.6	63.6	131.2	5486.5	133.73	211.25	353.3	3170.1	8831.7	0.02	0.62	0.36
24	AO-20%SO-2	AO-SO	120	1.99	2.23	2.5	188	44.3	64.8	128.7	5302.3	131.21	215.31	353.3	3323.6	8814.4	0.021	0.6	0.38
25	AO-20%SO-3	AO-SO	121	1.91	2.14	2.4	190	45.1	66.1	131.2	5256.7	133.73	215.31	353.3	3344.2	8791.2	0.022	0.6	0.38
26	AO-20%SO-4	AO-SO	120	1.84	2.06	2.31	200	45.1	64.8	126.3	5344.5	128.73	215.31	340.1	3367.8	8912.1	0.022	0.6	0.38
27	AO-20%SO-5	AO-SO	128	1.88	2.1	2.36	210	48.7	68.7	131.2	5177.2	133.73	223.67	360.1	3475.3	8862.6	0.024	0.58	0.39
28	AO-20%SO-6	AO-SO	120	1.88	2.1	2.36	195	45.1	64.8	126.3	5392.5	128.73	215.31	340.1	3340.1	8927.3	0.022	0.6	0.37
29	AO-20%SO-7	AO-SO	123	1.88	2.1	2.31	193	46	66.1	131.2	5337.4	133.73	219.45	353.3	3360	8890	0.022	0.6	0.38
30	AO-30%SO-1	AO-SO	119	2.02	2.27	2.54	192	42.6	64.8	128.7	5307.9	131.21	215.31	353.3	3258.2	8758.5	0.022	0.61	0.37
31	AO-30%SO-2	AO-SO	125	1.99	2.23	2.5	197	45.1	67.4	131.2	5152.2	133.73	219.45	367	3372.3	8721.7	0.023	0.59	0.39
32	AO-30%SO-3	AO-SO	121	1.91	2.14	2.4	205	44.3	64.8	128.7	5253.3	131.21	215.31	353.3	3346.7	8805.3	0.023	0.6	0.38
33	AO-30%SO-4	AO-SO	124	1.88	2.14	2.4	214	46	66.1	131.2	5170.5	133.73	219.45	360.1	3400.7	8784.9	0.024	0.59	0.39
34	AO-30%SO-5	AO-SO	128	1.91	2.18	2.45	220	46.9	68.7	133.7	5045.2	136.3	223.67	367	3426.1	8690.8	0.025	0.58	0.39
35	AO-30%SO-6	AO-SO	119	1.91	2.14	2.4	195	43.5	64.8	131.2	5374.6	133.73	215.31	353.3	3247.3	8816.5	0.022	0.61	0.37
36	AO-30%SO-7	AO-SO	125	1.99	2.23	2.5	198	45.1	67.4	133.7	5281.4	136.3	223.67	367	3302.5	8782.2	0.023	0.6	0.38
37	AO-40%SO-1	AO-SO	122	1.95	2.23	2.5	200	43.5	64.8	128.7	5220.6	131.21	219.45	360.1	3329.9	8750.6	0.023	0.6	0.38
38	AO-40%SO-2	AO-SO	125	1.99	2.23	2.5	204	45.1	66.1	131.2	5160.5	133.73	223.67	367	3362.9	8727.4	0.023	0.59	0.39
39	AO-40%SO-3	AO-SO	124	1.95	2.18	2.45	204	45.1	66.1	128.7	5155.1	131.21	219.45	360.1	3423.5	8782.8	0.023	0.59	0.39

40	AO-40%SO-4	AO-SO	128	1.84	2.1	2.36	210	46	67.4	131.2	5082.1	133.73	223.67	367	3468.2	8760.2	0.024	0.58	0.4
41	AO-40%SO-5	AO-SO	134	1.88	2.1	2.36	218	48.7	70	133.7	5016.6	136.3	232.36	374.1	3540.4	8775.4	0.025	0.57	0.4
42	AO-40%SO-6	AO-SO	123	2.02	2.27	2.54	192	44.3	66.1	131.2	5263.6	133.73	219.45	367	3306.3	8762.2	0.022	0.6	0.38
43	AO-40%SO-7	AO-SO	128	1.99	2.23	2.5	205	46	67.4	133.7	5175.5	136.3	227.97	374.1	3362.3	8742.9	0.023	0.59	0.39
44	AO-60%SO-1	AO-SO	126	1.99	2.23	2.54	202	44.3	66.1	131.2	5121.3	133.73	223.67	374.1	3397.2	8720.5	0.023	0.59	0.39
45	AO-60%SO-2	AO-SO	129	1.99	2.27	2.54	211	46	67.4	133.7	5057.4	136.3	227.97	381.3	3432.1	8700.9	0.024	0.58	0.39
46	AO-60%SO-3	AO-SO	134	1.99	2.27	2.54	210	47.8	68.7	133.7	4931.7	136.3	232.36	381.3	3536.2	8678	0.024	0.57	0.41
47	AO-60%SO-4	AO-SO	128	1.99	2.23	2.5	211	45.1	66.1	131.2	5071.2	133.73	227.97	374.1	3438.1	8720.6	0.024	0.58	0.39
48	AO-60%SO-5	AO-SO	133	1.99	2.23	2.5	215	46.9	68.7	133.7	4969.6	136.3	232.36	381.3	3501.2	8686.1	0.025	0.57	0.4
49	AO-60%SO-6	AO-SO	129	1.99	2.23	2.5	201	46	67.4	133.7	5129.9	136.3	227.97	381.3	3418.6	8749.7	0.023	0.59	0.39
50	AO-60%SO-7	AO-SO	134	1.95	2.18	2.45	211	47.8	68.7	136.3	5054.3	138.92	236.82	388.6	3454.5	8719.7	0.024	0.58	0.4
51	AO-80%SO-1	AO-SO	133	1.91	2.14	2.4	207	46.9	67.4	131.2	4994.3	133.73	232.36	381.3	3565.4	8766.2	0.024	0.57	0.41
52	AO-80%SO-2	AO-SO	137	1.91	2.14	2.4	213	47.8	68.7	133.7	4916.1	136.3	236.82	388.6	3614.4	8743.5	0.024	0.56	0.41
53	AO-80%SO-3	AO-SO	136	1.91	2.14	2.4	209	46.9	68.7	133.7	4958.5	136.3	236.82	388.6	3585.7	8752.8	0.024	0.57	0.41
54	AO-80%SO-4	AO-SO	140	1.91	2.14	2.4	218	49.7	70	136.3	4877.3	138.92	241.38	396.1	3634.6	8730.2	0.025	0.56	0.42
55	AO-80%SO-5	AO-SO	144	1.95	2.18	2.45	217	50.6	72.7	138.9	4789.1	141.6	246.02	411.5	3684.8	8690.6	0.025	0.55	0.42
56	AO-80%SO-6	AO-SO	134	1.99	2.23	2.5	201	46.9	68.7	136.3	5039.2	138.92	236.82	388.6	3496.5	8736.7	0.023	0.58	0.4
57	AO-80%SO-7	AO-SO	139	2.02	2.27	2.54	204	48.7	70	136.3	4894.5	138.92	241.38	396.1	3606.1	8704.3	0.023	0.56	0.41
58	AO-10%CO-1	AO-CO	120	2.1	2.31	2.59	190	44.3	66.1	131.2	5315.1	133.73	215.31	346.6	3226.8	8731.5	0.022	0.61	0.37
59	AO-10%CO-2	AO-CO	118	2.02	2.23	2.5	193	43.5	64.8	128.7	5361.2	131.21	211.25	346.6	3253.7	8807.8	0.022	0.61	0.37
60	AO-10%CO-3	AO-CO	120	2.1	2.31	2.59	190	44.3	66.1	131.2	5315.1	133.73	215.31	346.6	3226.8	8731.5	0.022	0.61	0.37

61	AO-10%CO-4	AO-CO	117	2.23	2.45	2.69	181	43.5	64.8	128.7	5341.3	131.21	211.25	346.6	3151.9	8674.1	0.021	0.62	0.36
62	AO-10%CO-5	AO-CO	124	2.23	2.45	2.75	187	46	67.4	133.7	5158.9	136.3	219.45	360.1	3268	8613.7	0.022	0.6	0.38
63	AO-10%CO-6	AO-CO	116	2.4	2.69	2.96	176	42.6	64.8	131.2	5333	133.73	211.25	346.6	3071.5	8580.5	0.021	0.62	0.36
64	AO-10%CO-7	AO-CO	122	2.4	2.64	2.96	182	45.1	67.4	133.7	5231.3	136.3	219.45	353.3	3133.4	8546.8	0.021	0.61	0.37
65	AO-20%CO-1	AO-CO	112	2.1	2.36	2.64	192	41	63.6	131.2	5511.6	133.73	207.26	346.6	3045	8748.1	0.022	0.63	0.35
66	AO-20%CO-2	AO-CO	123	2.1	2.36	2.64	202	45.1	67.4	133.7	5212.2	136.3	219.45	367	3271.1	8684.9	0.023	0.6	0.38
67	AO-20%CO-3	AO-CO	116	2.18	2.45	2.75	192	42.6	63.6	128.7	5333.4	131.21	211.25	346.6	3168.4	8693.6	0.022	0.61	0.36
68	AO-20%CO-4	AO-CO	123	2.31	2.59	2.85	188	45.1	67.4	133.7	5196.6	136.3	219.45	360.1	3205.3	8590.2	0.022	0.61	0.37
69	AO-20%CO-5	AO-CO	126	2.31	2.54	2.85	192	46	68.7	136.3	5096.4	138.92	223.67	367	3261.9	8550	0.022	0.6	0.38
70	AO-20%CO-6	AO-CO	119	2.31	2.54	2.85	168	43.5	66.1	131.2	5285.1	133.73	215.31	353.3	3150.1	8602.9	0.019	0.61	0.37
71	AO-20%CO-7	AO-CO	123	2.31	2.54	2.85	177	45.1	67.4	133.7	5170.2	136.3	219.45	360.1	3224	8570.8	0.021	0.6	0.38
72	AO-30%CO-1	AO-CO	117	2.14	2.4	2.69	192	41.8	63.6	131.2	5392	133.73	215.31	353.3	3144.1	8728.2	0.022	0.62	0.36
73	AO-30%CO-2	AO-CO	122	2.1	2.36	2.64	203	44.3	66.1	131.2	5220.1	133.73	219.45	360.1	3278.4	8701.2	0.023	0.6	0.38
74	AO-30%CO-3	AO-CO	126	2.1	2.36	2.64	211	46	67.4	133.7	5111.1	136.3	223.67	367	3350.9	8672.5	0.024	0.59	0.39
75	AO-30%CO-4	AO-CO	121	2.31	2.59	2.85	180	42.6	66.1	133.7	5284.9	136.3	219.45	360.1	3128.9	8593.4	0.021	0.62	0.36
76	AO-30%CO-5	AO-CO	129	2.31	2.59	2.91	189	46	68.7	136.3	5020.1	138.92	227.97	374.1	3313.4	8522.7	0.022	0.59	0.39
77	AO-30%CO-6	AO-CO	121	2.45	2.75	3.02	176	43.5	66.1	133.7	5199.2	136.3	219.45	360.1	3140.4	8515.4	0.021	0.61	0.37
78	AO-30%CO-7	AO-CO	126	2.4	2.69	3.02	185	45.1	68.7	136.3	5104.1	138.92	223.67	374.1	3196.1	8484.9	0.022	0.6	0.38
79	AO-40%CO-1	AO-CO	122	2.02	2.27	2.5	199	44.3	64.8	128.7	5285.7	131.21	219.45	353.3	3326.2	8811.4	0.023	0.6	0.38
80	AO-40%CO-2	AO-CO	128	2.06	2.27	2.54	201	46	67.4	131.2	5152.7	133.73	227.97	367	3414.7	8768.3	0.023	0.59	0.39
81	AO-40%CO-3	AO-CO	128	2.27	2.5	2.8	195	45.1	67.4	133.7	5124.2	136.3	227.97	374.1	3327.6	8646.6	0.023	0.59	0.39

82	AO-40%CO-4	AO-CO	129	2.27	2.54	2.85	181	45.1	68.7	136.3	5098.4	138.92	227.97	374.1	3312.8	8592.2	0.021	0.59	0.39
83	AO-40%CO-5	AO-CO	134	2.27	2.54	2.8	189	47.8	70	136.3	4940.5	138.92	232.36	388.6	3430	8559.8	0.022	0.58	0.4
84	AO-40%CO-6	AO-CO	125	2.5	2.75	3.08	175	44.3	67.4	133.7	5132.4	136.3	223.67	367	3228.9	8536.1	0.02	0.6	0.38
85	AO-40%CO-7	AO-CO	129	2.45	2.69	3.02	180	46	68.7	136.3	5030.2	138.92	227.97	381.3	3292	8501.9	0.021	0.59	0.39
86	AO-60%CO-1	AO-CO	122	2.14	2.4	2.69	198	42.6	64.8	133.7	5275.3	136.3	223.67	367	3237.2	8710.5	0.023	0.61	0.37
87	AO-60%CO-2	AO-CO	135	2.18	2.45	2.75	203	46.9	70	136.3	4963.2	138.92	236.82	388.6	3458.7	8625.1	0.024	0.58	0.4
88	AO-60%CO-3	AO-CO	126	2.31	2.59	2.91	198	43.5	66.1	133.7	5108.7	136.3	227.97	374.1	3282.2	8589.1	0.023	0.6	0.38
89	AO-60%CO-4	AO-CO	128	2.4	2.64	2.96	181	43.5	67.4	133.7	5088.5	136.3	227.97	374.1	3287.1	8556.2	0.021	0.6	0.38
90	AO-60%CO-5	AO-CO	132	2.36	2.64	2.96	190	45.1	68.7	136.3	4975.7	138.92	232.36	388.6	3359.9	8525.3	0.022	0.58	0.39
91	AO-60%CO-6	AO-CO	131	2.54	2.85	3.2	183	44.3	68.7	136.3	4982.4	138.92	232.36	388.6	3277.1	8442.4	0.022	0.59	0.39
92	AO-60%CO-7	AO-CO	135	2.5	2.8	3.14	184	45.1	70	138.9	4886.2	141.6	236.82	396.1	3343.9	8414.1	0.022	0.58	0.4
93	AO-80%CO-1	AO-CO	127	2.18	2.45	2.75	197	42.6	66.1	133.7	5119.7	136.3	227.97	381.3	3337.9	8654.1	0.023	0.59	0.39
94	AO-80%CO-2	AO-CO	137	2.23	2.5	2.8	208	46	70	136.3	4836	138.92	236.82	403.7	3532.5	8576.2	0.024	0.56	0.41
95	AO-80%CO-3	AO-CO	131	2.36	2.64	2.96	196	43.5	68.7	136.3	5007.3	138.92	232.36	388.6	3346.1	8548.9	0.023	0.59	0.39
96	AO-80%CO-4	AO-CO	136	2.36	2.64	2.96	202	45.1	70	136.3	4850.2	138.92	236.82	396.1	3463.6	8516.2	0.024	0.57	0.41
97	AO-80%CO-5	AO-CO	136	2.5	2.8	3.14	192	45.1	70	136.3	4789.8	138.92	236.82	396.1	3429.2	8410.8	0.023	0.57	0.41
98	AO-80%CO-6	AO-CO	141	2.54	2.85	3.2	195	46.9	71.3	138.9	4689.2	141.6	241.38	403.7	3496.1	8379.8	0.023	0.56	0.42
99	AO-80%CO-7	AO-CO	140	2.54	2.85	3.2	186	46	71.3	138.9	4726.9	141.6	241.38	403.7	3430.4	8343.8	0.022	0.57	0.41
100	AO-10%RO-1	AO-RO	117	2.75	3.02	3.38	152	42.6	66.1	133.7	5219.5	136.3	211.25	353.3	3013.6	8384.7	0.018	0.62	0.36
101	AO-10%RO-2	AO-RO	119	2.5	2.8	3.08	166	43.5	66.1	133.7	5266.2	136.3	215.31	360.1	3064.8	8497.2	0.02	0.62	0.36
102	AO-10%RO-3	AO-RO	121	2.69	2.96	3.32	173	43.5	67.4	133.7	5093.7	136.3	215.31	360.1	3086.8	8353.3	0.021	0.61	0.37

103	AO-10%RO-4	AO-RO	117	2.75	3.02	3.38	152	42.6	66.1	133.7	5219.5	136.3	211.25	353.3	3013.6	8384.7	0.018	0.62	0.36
104	AO-10%RO-5	AO-RO	128	2.64	2.91	3.26	157	46.9	70	136.3	4933.5	138.92	223.67	367	3232.8	8323.2	0.019	0.59	0.39
105	AO-10%RO-6	AO-RO	117	2.69	3.02	3.32	151	43.5	66.1	133.7	5192.4	136.3	211.25	353.3	3018.3	8361.8	0.018	0.62	0.36
106	AO-10%RO-7	AO-RO	124	2.75	3.02	3.32	154	45.1	67.4	133.7	5030	136.3	219.45	360.1	3143.8	8328	0.019	0.6	0.38
107	AO-20%RO-1	AO-RO	115	2.5	2.8	3.08	164	41	64.8	131.2	5387.9	133.73	211.25	346.6	2981.3	8533	0.019	0.63	0.35
108	AO-20%RO-2	AO-RO	119	2.45	2.75	3.02	171	43.5	66.1	133.7	5261.4	136.3	215.31	360.1	3067.5	8499.7	0.02	0.62	0.36
109	AO-20%RO-3	AO-RO	117	2.54	2.8	3.14	160	42.6	66.1	133.7	5274.2	136.3	211.25	353.3	3013	8447.7	0.019	0.62	0.36
110	AO-20%RO-4	AO-RO	124	2.64	2.96	3.26	160	45.1	67.4	133.7	5016.6	136.3	219.45	367	3163.3	8339.6	0.019	0.6	0.38
111	AO-20%RO-5	AO-RO	129	2.59	2.85	3.2	162	46	70	136.3	4908.2	138.92	223.67	374.1	3240.8	8311.1	0.02	0.59	0.39
112	AO-20%RO-6	AO-RO	119	2.85	3.2	3.52	143	42.6	67.4	133.7	5119.3	136.3	211.25	360.1	3015.2	8278	0.017	0.62	0.36
113	AO-20%RO-7	AO-RO	124	2.85	3.14	3.52	151	44.3	68.7	136.3	5018.9	138.92	219.45	367	3081.2	8251.4	0.018	0.61	0.37
114	AO-30%RO-1	AO-RO	115	2.5	2.8	3.14	163	41	64.8	131.2	5356.6	133.73	211.25	353.3	3009.5	8528.8	0.019	0.63	0.35
115	AO-30%RO-2	AO-RO	126	2.5	2.8	3.08	171	45.1	68.7	136.3	5107.2	138.92	223.67	367	3173.7	8452.2	0.02	0.6	0.38
116	AO-30%RO-3	AO-RO	119	2.64	2.96	3.26	159	42.6	66.1	133.7	5243.4	136.3	215.31	360.1	3031.9	8434.5	0.019	0.62	0.36
117	AO-30%RO-4	AO-RO	119	2.64	2.96	3.26	159	42.6	66.1	133.7	5243.4	136.3	215.31	360.1	3031.9	8434.5	0.019	0.62	0.36
118	AO-30%RO-5	AO-RO	124	2.69	2.96	3.32	162	44.3	67.4	133.7	5062.3	136.3	219.45	367	3173.5	8397.6	0.019	0.6	0.38
119	AO-30%RO-6	AO-RO	117	2.8	3.14	3.52	150	41	66.1	133.7	5221.1	136.3	211.25	360.1	2969.8	8340.8	0.018	0.63	0.36
120	AO-30%RO-7	AO-RO	124	2.85	3.14	3.45	152	44.3	68.7	136.3	5007.1	138.92	219.45	374.1	3138.6	8297.7	0.018	0.6	0.38
121	AO-40%RO-1	AO-RO	116	2.59	2.91	3.26	167	41	66.1	133.7	5334.3	136.3	211.25	360.1	2975.9	8477.7	0.02	0.63	0.35
122	AO-40%RO-2	AO-RO	121	2.54	2.85	3.2	172	43.5	67.4	136.3	5203.5	138.92	219.45	367	3066.8	8442.6	0.02	0.62	0.36
123	AO-40%RO-3	AO-RO	119	2.69	3.02	3.32	159	42.6	66.1	133.7	5189.3	136.3	215.31	360.1	3055	8403.2	0.019	0.62	0.36

124	AO-40%RO-4	AO-RO	124	2.59	2.91	3.2	166	44.3	68.7	136.3	5072.5	138.92	219.45	374.1	3130	8368.9	0.02	0.61	0.37
125	AO-40%RO-5	AO-RO	129	2.69	2.96	3.26	161	46	70	136.3	4866.8	138.92	223.67	381.3	3278.3	8306.5	0.019	0.59	0.4
126	AO-40%RO-6	AO-RO	120	2.96	3.26	3.65	148	42.6	67.4	136.3	5142.6	138.92	215.31	367	3018.6	8309.5	0.018	0.62	0.36
127	AO-40%RO-7	AO-RO	125	2.85	3.2	3.52	152	44.3	68.7	136.3	4964.2	138.92	219.45	374.1	3147.7	8263.6	0.018	0.6	0.38
128	AO-60%RO-1	AO-RO	115	2.64	2.96	3.32	168	40.3	66.1	136.3	5366	138.92	211.25	367	2910.9	8444.6	0.02	0.64	0.35
129	AO-60%RO-2	AO-RO	122	2.59	2.91	3.2	171	43.5	67.4	136.3	5173.6	138.92	219.45	367	3069.7	8414.2	0.02	0.62	0.37
130	AO-60%RO-3	AO-RO	120	2.75	3.08	3.45	162	42.6	67.4	136.3	5182.3	138.92	215.31	367	3037	8380.9	0.019	0.62	0.36
131	AO-60%RO-4	AO-RO	125	2.64	2.96	3.32	164	44.3	68.7	138.9	5070	141.6	223.67	381.3	3115.4	8349.1	0.02	0.61	0.37
132	AO-60%RO-5	AO-RO	127	2.75	3.08	3.45	163	44.3	68.7	136.3	4965.6	138.92	223.67	374.1	3179.5	8308.4	0.02	0.6	0.38
133	AO-60%RO-6	AO-RO	131	2.75	3.08	3.38	162	46	71.3	138.9	4865	141.6	227.97	388.6	3246.3	8273.3	0.02	0.59	0.39
134	AO-60%RO-7	AO-RO	123	2.91	3.2	3.58	153	43.5	68.7	138.9	5037	141.6	219.45	381.3	3066.6	8256.5	0.019	0.61	0.37
135	AO-80%RO-1	AO-RO	117	2.69	3.02	3.38	172	40.3	66.1	136.3	5308.6	138.92	215.31	367	2913.3	8394	0.021	0.63	0.35
136	AO-80%RO-2	AO-RO	127	2.75	3.08	3.45	176	44.3	70	138.9	4993.1	141.6	223.67	381.3	3145.6	8314.8	0.021	0.6	0.38
137	AO-80%RO-3	AO-RO	121	2.85	3.2	3.58	178	41.8	68.7	138.9	5117.3	141.6	219.45	374.1	2931.8	8227.4	0.022	0.62	0.36
138	AO-80%RO-4	AO-RO	126	2.8	3.14	3.52	183	43.5	70	138.9	4948.5	141.6	223.67	388.6	3062.2	8193.7	0.022	0.6	0.37
139	AO-80%RO-5	AO-RO	126	2.85	3.14	3.52	168	43.5	68.7	136.3	4935	138.92	223.67	381.3	3149.7	8252.6	0.02	0.6	0.38
140	AO-80%RO-6	AO-RO	132	2.85	3.14	3.52	166	45.1	71.3	138.9	4825.1	141.6	227.97	388.6	3231.2	8222.5	0.02	0.59	0.39
141	AO-80%RO-7	AO-RO	132	2.85	3.2	3.52	157	45.1	71.3	138.9	4819.4	141.6	227.97	388.6	3218.8	8195.8	0.019	0.59	0.39

Table S4. Data used for the establishment of SIMCA model.

Cal/Val	Number	Name	Group	T2W	T21S	T21P	T21E	S21	T22S	T22P	T22E	S22	T23S	T23P	T23E	S23	STotal	P21	P22	P23
Calibration	1	AO-1	AO	115	2.8	3.14	3.45	141	41.84	64.84	134	5317	136	211.2	353.3	2905.5	8363.1	0.017	0.64	0.347
	2	AO-2	AO	120	2.8	3.08	3.45	138	43.46	67.36	134	5136	136	215.3	360.1	3046.8	8320.6	0.017	0.62	0.366
	3	AO-3	AO	126	2.69	2.96	3.32	146	45.15	68.65	136	5007	139	223.7	374.1	3140.8	8293.1	0.018	0.6	0.379
	4	AO-4	AO	119	2.85	3.14	3.52	149	43.46	66.09	134	5102	136	215.3	360.1	3016.7	8267.3	0.018	0.62	0.365
	5	AO-5	AO	124	2.85	3.14	3.52	148	45.15	68.65	136	4989	139	219.4	374.1	3097.2	8233.9	0.018	0.61	0.376
	6	AO-6	AO	115	2.91	3.2	3.58	140	41.84	64.84	134	5271	136	211.2	353.3	2884.5	8295.9	0.017	0.64	0.348
	7	AO-7	AO	126	2.8	3.08	3.45	145	45.15	68.65	136	4976	139	223.7	374.1	3112.4	8233.3	0.018	0.6	0.378
	8	AO-8	AO	124	2.85	3.14	3.52	140	44.3	67.36	134	5015	136	219.4	367	3122.3	8277.5	0.017	0.61	0.377
	9	AO-9	AO	128	2.75	3.08	3.38	142	46.02	69.97	136	4934	139	223.7	374.1	3178.6	8254.9	0.017	0.6	0.385
	10	AO-10	AO	123	2.8	3.08	3.45	143	44.3	68.65	136	5063	139	219.4	367	3054.6	8261	0.017	0.61	0.37
	11	AO-11	AO	128	2.75	3.08	3.38	146	46.02	69.97	136	4915	139	223.7	374.1	3173.6	8234.5	0.018	0.6	0.385
	12	AO-12	AO	123	2.91	3.26	3.65	142	44.3	68.65	136	5037	139	219.4	367	3035.8	8214.7	0.017	0.61	0.37
	13	AO-13	AO	118	2.85	3.14	3.52	133	42.64	66.09	134	5168	136	211.2	360.1	2999.8	8300.2	0.016	0.62	0.361
	14	AO-14	AO	125	2.8	3.08	3.38	141	45.15	68.65	134	4998	136	219.4	360.1	3131	8269.9	0.017	0.6	0.379
	15	AO-15	AO	129	2.8	3.08	3.45	144	46.9	69.97	136	4887	139	223.7	374.1	3210.7	8241.1	0.017	0.59	0.39
	16	AO-10%SO-1	AO-SO	112	2.06	2.36	2.69	174	41.05	64.84	131	5488	134	203.3	346.6	3071.1	8733.3	0.02	0.63	0.352
	17	AO-10%SO-2	AO-SO	115	2.06	2.36	2.69	183	41.84	64.84	131	5371	134	207.3	353.3	3143.9	8698.5	0.021	0.62	0.361
	18	AO-10%SO-3	AO-SO	114	1.91	2.14	2.4	199	43.46	63.62	129	5441	131	207.3	340.1	3192.2	8832.3	0.023	0.62	0.361

19	AO-10%SO-4	AO-SO	116	1.91	2.18	2.45	200	43.46	64.84	129	5384	131	207.3	346.6	3225.6	8809.3	0.023	0.61	0.366
20	AO-10%SO-5	AO-SO	125	1.95	2.18	2.45	216	46.9	67.36	131	5153	134	219.4	360.1	3391	8760.2	0.025	0.59	0.387
21	AO-10%SO-6	AO-SO	114	1.91	2.1	2.36	195	43.46	63.62	129	5471	131	207.3	340.1	3190.7	8856.2	0.022	0.62	0.36
22	AO-10%SO-7	AO-SO	121	1.88	2.1	2.36	209	45.15	66.09	129	5296	131	215.3	346.6	3326.3	8831.8	0.024	0.6	0.377
23	AO-20%SO-1	AO-SO	115	1.95	2.18	2.45	175	42.64	63.62	131	5486	134	211.2	353.3	3170.1	8831.7	0.02	0.62	0.359
24	AO-20%SO-2	AO-SO	120	1.99	2.23	2.5	188	44.3	64.84	129	5302	131	215.3	353.3	3323.6	8814.4	0.021	0.6	0.377
25	AO-20%SO-3	AO-SO	121	1.91	2.14	2.4	190	45.15	66.09	131	5257	134	215.3	353.3	3344.2	8791.2	0.022	0.6	0.38
26	AO-20%SO-4	AO-SO	120	1.84	2.06	2.31	200	45.15	64.84	126	5344	129	215.3	340.1	3367.8	8912.1	0.022	0.6	0.378
27	AO-20%SO-5	AO-SO	128	1.88	2.1	2.36	210	48.72	68.65	131	5177	134	223.7	360.1	3475.3	8862.6	0.024	0.58	0.392
28	AO-20%SO-6	AO-SO	120	1.88	2.1	2.36	195	45.15	64.84	126	5392	129	215.3	340.1	3340.1	8927.3	0.022	0.6	0.374
29	AO-20%SO-7	AO-SO	123	1.88	2.1	2.31	193	46.02	66.09	131	5337	134	219.4	353.3	3360	8890	0.022	0.6	0.378
30	AO-30%SO-1	AO-SO	119	2.02	2.27	2.54	192	42.64	64.84	129	5308	131	215.3	353.3	3258.2	8758.5	0.022	0.61	0.372
31	AO-30%SO-2	AO-SO	125	1.99	2.23	2.5	197	45.15	67.36	131	5152	134	219.4	367	3372.3	8721.7	0.023	0.59	0.387
32	AO-30%SO-3	AO-SO	121	1.91	2.14	2.4	205	44.3	64.84	129	5253	131	215.3	353.3	3346.7	8805.3	0.023	0.6	0.38
33	AO-30%SO-4	AO-SO	124	1.88	2.14	2.4	214	46.02	66.09	131	5171	134	219.4	360.1	3400.7	8784.9	0.024	0.59	0.387
34	AO-30%SO-5	AO-SO	128	1.91	2.18	2.45	220	46.9	68.65	134	5045	136	223.7	367	3426.1	8690.8	0.025	0.58	0.394
35	AO-30%SO-6	AO-SO	119	1.91	2.14	2.4	195	43.46	64.84	131	5375	134	215.3	353.3	3247.3	8816.5	0.022	0.61	0.368
36	AO-30%SO-7	AO-SO	125	1.99	2.23	2.5	198	45.15	67.36	134	5281	136	223.7	367	3302.5	8782.2	0.023	0.6	0.376
37	AO-40%SO-1	AO-SO	122	1.95	2.23	2.5	200	43.46	64.84	129	5221	131	219.4	360.1	3329.9	8750.6	0.023	0.6	0.381
38	AO-40%SO-2	AO-SO	125	1.99	2.23	2.5	204	45.15	66.09	131	5161	134	223.7	367	3362.9	8727.4	0.023	0.59	0.385
39	AO-40%SO-3	AO-SO	124	1.95	2.18	2.45	204	45.15	66.09	129	5155	131	219.4	360.1	3423.5	8782.8	0.023	0.59	0.39

40	AO-40%SO-4	AO-SO	128	1.84	2.1	2.36	210	46.02	67.36	131	5082	134	223.7	367	3468.2	8760.2	0.024	0.58	0.396
41	AO-40%SO-5	AO-SO	134	1.88	2.1	2.36	218	48.72	69.97	134	5017	136	232.4	374.1	3540.4	8775.4	0.025	0.57	0.403
42	AO-40%SO-6	AO-SO	123	2.02	2.27	2.54	192	44.3	66.09	131	5264	134	219.4	367	3306.3	8762.2	0.022	0.6	0.377
43	AO-40%SO-7	AO-SO	128	1.99	2.23	2.5	205	46.02	67.36	134	5175	136	228	374.1	3362.3	8742.9	0.023	0.59	0.385
44	AO-60%SO-1	AO-SO	126	1.99	2.23	2.54	202	44.3	66.09	131	5121	134	223.7	374.1	3397.2	8720.5	0.023	0.59	0.39
45	AO-60%SO-2	AO-SO	129	1.99	2.27	2.54	211	46.02	67.36	134	5057	136	228	381.3	3432.1	8700.9	0.024	0.58	0.394
46	AO-60%SO-3	AO-SO	134	1.99	2.27	2.54	210	47.81	68.65	134	4932	136	232.4	381.3	3536.2	8678	0.024	0.57	0.407
47	AO-60%SO-4	AO-SO	128	1.99	2.23	2.5	211	45.15	66.09	131	5071	134	228	374.1	3438.1	8720.6	0.024	0.58	0.394
48	AO-60%SO-5	AO-SO	133	1.99	2.23	2.5	215	46.9	68.65	134	4970	136	232.4	381.3	3501.2	8686.1	0.025	0.57	0.403
49	AO-60%SO-6	AO-SO	129	1.99	2.23	2.5	201	46.02	67.36	134	5130	136	228	381.3	3418.6	8749.7	0.023	0.59	0.391
50	AO-60%SO-7	AO-SO	134	1.95	2.18	2.45	211	47.81	68.65	136	5054	139	236.8	388.6	3454.5	8719.7	0.024	0.58	0.396
51	AO-80%SO-1	AO-SO	133	1.91	2.14	2.4	207	46.9	67.36	131	4994	134	232.4	381.3	3565.4	8766.2	0.024	0.57	0.407
52	AO-80%SO-2	AO-SO	137	1.91	2.14	2.4	213	47.81	68.65	134	4916	136	236.8	388.6	3614.4	8743.5	0.024	0.56	0.413
53	AO-80%SO-3	AO-SO	136	1.91	2.14	2.4	209	46.9	68.65	134	4958	136	236.8	388.6	3585.7	8752.8	0.024	0.57	0.41
54	AO-80%SO-4	AO-SO	140	1.91	2.14	2.4	218	49.66	69.97	136	4877	139	241.4	396.1	3634.6	8730.2	0.025	0.56	0.416
55	AO-80%SO-5	AO-SO	144	1.95	2.18	2.45	217	50.62	72.69	139	4789	142	246	411.5	3684.8	8690.6	0.025	0.55	0.424
56	AO-80%SO-6	AO-SO	134	1.99	2.23	2.5	201	46.9	68.65	136	5039	139	236.8	388.6	3496.5	8736.7	0.023	0.58	0.4
57	AO-80%SO-7	AO-SO	139	2.02	2.27	2.54	204	48.72	69.97	136	4894	139	241.4	396.1	3606.1	8704.3	0.023	0.56	0.414
58	AO-10%CO-1	AO-CO	120	2.1	2.31	2.59	190	44.3	66.09	131	5315	134	215.3	346.6	3226.8	8731.5	0.022	0.61	0.37
59	AO-10%CO-2	AO-CO	118	2.02	2.23	2.5	193	43.46	64.84	129	5361	131	211.2	346.6	3253.7	8807.8	0.022	0.61	0.369
60	AO-10%CO-3	AO-CO	120	2.1	2.31	2.59	190	44.3	66.09	131	5315	134	215.3	346.6	3226.8	8731.5	0.022	0.61	0.37

61	AO-10%CO-4	AO-CO	117	2.23	2.45	2.69	181	43.46	64.84	129	5341	131	211.2	346.6	3151.9	8674.1	0.021	0.62	0.363
62	AO-10%CO-5	AO-CO	124	2.23	2.45	2.75	187	46.02	67.36	134	5159	136	219.4	360.1	3268	8613.7	0.022	0.6	0.379
63	AO-10%CO-6	AO-CO	116	2.4	2.69	2.96	176	42.64	64.84	131	5333	134	211.2	346.6	3071.5	8580.5	0.021	0.62	0.358
64	AO-10%CO-7	AO-CO	122	2.4	2.64	2.96	182	45.15	67.36	134	5231	136	219.4	353.3	3133.4	8546.8	0.021	0.61	0.367
65	AO-20%CO-1	AO-CO	112	2.1	2.36	2.64	192	41.05	63.62	131	5512	134	207.3	346.6	3045	8748.1	0.022	0.63	0.348
66	AO-20%CO-2	AO-CO	123	2.1	2.36	2.64	202	45.15	67.36	134	5212	136	219.4	367	3271.1	8684.9	0.023	0.6	0.377
67	AO-20%CO-3	AO-CO	116	2.18	2.45	2.75	192	42.64	63.62	129	5333	131	211.2	346.6	3168.4	8693.6	0.022	0.61	0.364
68	AO-20%CO-4	AO-CO	123	2.31	2.59	2.85	188	45.15	67.36	134	5197	136	219.4	360.1	3205.3	8590.2	0.022	0.61	0.373
69	AO-20%CO-5	AO-CO	126	2.31	2.54	2.85	192	46.02	68.65	136	5096	139	223.7	367	3261.9	8550	0.022	0.6	0.382
70	AO-20%CO-6	AO-CO	119	2.31	2.54	2.85	168	43.46	66.09	131	5285	134	215.3	353.3	3150.1	8602.9	0.019	0.61	0.366
71	AO-20%CO-7	AO-CO	123	2.31	2.54	2.85	177	45.15	67.36	134	5170	136	219.4	360.1	3224	8570.8	0.021	0.6	0.376
72	AO-30%CO-1	AO-CO	117	2.14	2.4	2.69	192	41.84	63.62	131	5392	134	215.3	353.3	3144.1	8728.2	0.022	0.62	0.36
73	AO-30%CO-2	AO-CO	122	2.1	2.36	2.64	203	44.3	66.09	131	5220	134	219.4	360.1	3278.4	8701.2	0.023	0.6	0.377
74	AO-30%CO-3	AO-CO	126	2.1	2.36	2.64	211	46.02	67.36	134	5111	136	223.7	367	3350.9	8672.5	0.024	0.59	0.386
75	AO-30%CO-4	AO-CO	121	2.31	2.59	2.85	180	42.64	66.09	134	5285	136	219.4	360.1	3128.9	8593.4	0.021	0.62	0.364
76	AO-30%CO-5	AO-CO	129	2.31	2.59	2.91	189	46.02	68.65	136	5020	139	228	374.1	3313.4	8522.7	0.022	0.59	0.389
77	AO-30%CO-6	AO-CO	121	2.45	2.75	3.02	176	43.46	66.09	134	5199	136	219.4	360.1	3140.4	8515.4	0.021	0.61	0.369
78	AO-30%CO-7	AO-CO	126	2.4	2.69	3.02	185	45.15	68.65	136	5104	139	223.7	374.1	3196.1	8484.9	0.022	0.6	0.377
79	AO-40%CO-1	AO-CO	122	2.02	2.27	2.5	199	44.3	64.84	129	5286	131	219.4	353.3	3326.2	8811.4	0.023	0.6	0.377
80	AO-40%CO-2	AO-CO	128	2.06	2.27	2.54	201	46.02	67.36	131	5153	134	228	367	3414.7	8768.3	0.023	0.59	0.389
81	AO-40%CO-3	AO-CO	128	2.27	2.5	2.8	195	45.15	67.36	134	5124	136	228	374.1	3327.6	8646.6	0.023	0.59	0.385

82	AO-40%CO-4	AO-CO	129	2.27	2.54	2.85	181	45.15	68.65	136	5098	139	228	374.1	3312.8	8592.2	0.021	0.59	0.386
83	AO-40%CO-5	AO-CO	134	2.27	2.54	2.8	189	47.81	69.97	136	4940	139	232.4	388.6	3430	8559.8	0.022	0.58	0.401
84	AO-40%CO-6	AO-CO	125	2.5	2.75	3.08	175	44.3	67.36	134	5132	136	223.7	367	3228.9	8536.1	0.02	0.6	0.378
85	AO-40%CO-7	AO-CO	129	2.45	2.69	3.02	180	46.02	68.65	136	5030	139	228	381.3	3292	8501.9	0.021	0.59	0.387
86	AO-60%CO-1	AO-CO	122	2.14	2.4	2.69	198	42.64	64.84	134	5275	136	223.7	367	3237.2	8710.5	0.023	0.61	0.372
87	AO-60%CO-2	AO-CO	135	2.18	2.45	2.75	203	46.9	69.97	136	4963	139	236.8	388.6	3458.7	8625.1	0.024	0.58	0.401
88	AO-60%CO-3	AO-CO	126	2.31	2.59	2.91	198	43.46	66.09	134	5109	136	228	374.1	3282.2	8589.1	0.023	0.6	0.382
89	AO-60%CO-4	AO-CO	128	2.4	2.64	2.96	181	43.46	67.36	134	5088	136	228	374.1	3287.1	8556.2	0.021	0.6	0.384
90	AO-60%CO-5	AO-CO	132	2.36	2.64	2.96	190	45.15	68.65	136	4976	139	232.4	388.6	3359.9	8525.3	0.022	0.58	0.394
91	AO-60%CO-6	AO-CO	131	2.54	2.85	3.2	183	44.3	68.65	136	4982	139	232.4	388.6	3277.1	8442.4	0.022	0.59	0.388
92	AO-60%CO-7	AO-CO	135	2.5	2.8	3.14	184	45.15	69.97	139	4886	142	236.8	396.1	3343.9	8414.1	0.022	0.58	0.397
93	AO-80%CO-1	AO-CO	127	2.18	2.45	2.75	197	42.64	66.09	134	5120	136	228	381.3	3337.9	8654.1	0.023	0.59	0.386
94	AO-80%CO-2	AO-CO	137	2.23	2.5	2.8	208	46.02	69.97	136	4836	139	236.8	403.7	3532.5	8576.2	0.024	0.56	0.412
95	AO-80%CO-3	AO-CO	131	2.36	2.64	2.96	196	43.46	68.65	136	5007	139	232.4	388.6	3346.1	8548.9	0.023	0.59	0.391
96	AO-80%CO-4	AO-CO	136	2.36	2.64	2.96	202	45.15	69.97	136	4850	139	236.8	396.1	3463.6	8516.2	0.024	0.57	0.407
97	AO-80%CO-5	AO-CO	136	2.5	2.8	3.14	192	45.15	69.97	136	4790	139	236.8	396.1	3429.2	8410.8	0.023	0.57	0.408
98	AO-80%CO-6	AO-CO	141	2.54	2.85	3.2	195	46.9	71.32	139	4689	142	241.4	403.7	3496.1	8379.8	0.023	0.56	0.417
99	AO-80%CO-7	AO-CO	140	2.54	2.85	3.2	186	46.02	71.32	139	4727	142	241.4	403.7	3430.4	8343.8	0.022	0.57	0.411
100	AO-10%RO-1	AO-RO	117	2.75	3.02	3.38	152	42.64	66.09	134	5219	136	211.2	353.3	3013.6	8384.7	0.018	0.62	0.359
101	AO-10%RO-2	AO-RO	119	2.5	2.8	3.08	166	43.46	66.09	134	5266	136	215.3	360.1	3064.8	8497.2	0.02	0.62	0.361
102	AO-10%RO-3	AO-RO	121	2.69	2.96	3.32	173	43.46	67.36	134	5094	136	215.3	360.1	3086.8	8353.3	0.021	0.61	0.37

103	AO-10%RO-4	AO-RO	117	2.75	3.02	3.38	152	42.64	66.09	134	5219	136	211.2	353.3	3013.6	8384.7	0.018	0.62	0.359
104	AO-10%RO-5	AO-RO	128	2.64	2.91	3.26	157	46.9	69.97	136	4933	139	223.7	367	3232.8	8323.2	0.019	0.59	0.388
105	AO-10%RO-6	AO-RO	117	2.69	3.02	3.32	151	43.46	66.09	134	5192	136	211.2	353.3	3018.3	8361.8	0.018	0.62	0.361
106	AO-10%RO-7	AO-RO	124	2.75	3.02	3.32	154	45.15	67.36	134	5030	136	219.4	360.1	3143.8	8328	0.019	0.6	0.378
107	AO-20%RO-1	AO-RO	115	2.5	2.8	3.08	164	41.05	64.84	131	5388	134	211.2	346.6	2981.3	8533	0.019	0.63	0.349
108	AO-20%RO-2	AO-RO	119	2.45	2.75	3.02	171	43.46	66.09	134	5261	136	215.3	360.1	3067.5	8499.7	0.02	0.62	0.361
109	AO-20%RO-3	AO-RO	117	2.54	2.8	3.14	160	42.64	66.09	134	5274	136	211.2	353.3	3013	8447.7	0.019	0.62	0.357
110	AO-20%RO-4	AO-RO	124	2.64	2.96	3.26	160	45.15	67.36	134	5017	136	219.4	367	3163.3	8339.6	0.019	0.6	0.379
111	AO-20%RO-5	AO-RO	129	2.59	2.85	3.2	162	46.02	69.97	136	4908	139	223.7	374.1	3240.8	8311.1	0.02	0.59	0.39
112	AO-20%RO-6	AO-RO	119	2.85	3.2	3.52	143	42.64	67.36	134	5119	136	211.2	360.1	3015.2	8278	0.017	0.62	0.364
113	AO-20%RO-7	AO-RO	124	2.85	3.14	3.52	151	44.3	68.65	136	5019	139	219.4	367	3081.2	8251.4	0.018	0.61	0.373
114	AO-30%RO-1	AO-RO	115	2.5	2.8	3.14	163	41.05	64.84	131	5357	134	211.2	353.3	3009.5	8528.8	0.019	0.63	0.353
115	AO-30%RO-2	AO-RO	126	2.5	2.8	3.08	171	45.15	68.65	136	5107	139	223.7	367	3173.7	8452.2	0.02	0.6	0.375
116	AO-30%RO-3	AO-RO	119	2.64	2.96	3.26	159	42.64	66.09	134	5243	136	215.3	360.1	3031.9	8434.5	0.019	0.62	0.359
117	AO-30%RO-4	AO-RO	119	2.64	2.96	3.26	159	42.64	66.09	134	5243	136	215.3	360.1	3031.9	8434.5	0.019	0.62	0.359
118	AO-30%RO-5	AO-RO	124	2.69	2.96	3.32	162	44.3	67.36	134	5062	136	219.4	367	3173.5	8397.6	0.019	0.6	0.378
119	AO-30%RO-6	AO-RO	117	2.8	3.14	3.52	150	41.05	66.09	134	5221	136	211.2	360.1	2969.8	8340.8	0.018	0.63	0.356
120	AO-30%RO-7	AO-RO	124	2.85	3.14	3.45	152	44.3	68.65	136	5007	139	219.4	374.1	3138.6	8297.7	0.018	0.6	0.378
121	AO-40%RO-1	AO-RO	116	2.59	2.91	3.26	167	41.05	66.09	134	5334	136	211.2	360.1	2975.9	8477.7	0.02	0.63	0.351
122	AO-40%RO-2	AO-RO	121	2.54	2.85	3.2	172	43.46	67.36	136	5203	139	219.4	367	3066.8	8442.6	0.02	0.62	0.363
123	AO-40%RO-3	AO-RO	119	2.69	3.02	3.32	159	42.64	66.09	134	5189	136	215.3	360.1	3055	8403.2	0.019	0.62	0.364

	124	AO-40%RO-4	AO-RO	124	2.59	2.91	3.2	166	44.3	68.65	136	5073	139	219.4	374.1	3130	8368.9	0.02	0.61	0.374
	125	AO-40%RO-5	AO-RO	129	2.69	2.96	3.26	161	46.02	69.97	136	4867	139	223.7	381.3	3278.3	8306.5	0.019	0.59	0.395
	126	AO-40%RO-6	AO-RO	120	2.96	3.26	3.65	148	42.64	67.36	136	5143	139	215.3	367	3018.6	8309.5	0.018	0.62	0.363
	127	AO-40%RO-7	AO-RO	125	2.85	3.2	3.52	152	44.3	68.65	136	4964	139	219.4	374.1	3147.7	8263.6	0.018	0.6	0.381
	128	AO-60%RO-1	AO-RO	115	2.64	2.96	3.32	168	40.27	66.09	136	5366	139	211.2	367	2910.9	8444.6	0.02	0.64	0.345
	129	AO-60%RO-2	AO-RO	122	2.59	2.91	3.2	171	43.46	67.36	136	5174	139	219.4	367	3069.7	8414.2	0.02	0.62	0.365
	130	AO-60%RO-3	AO-RO	120	2.75	3.08	3.45	162	42.64	67.36	136	5182	139	215.3	367	3037	8380.9	0.019	0.62	0.362
	131	AO-60%RO-4	AO-RO	125	2.64	2.96	3.32	164	44.3	68.65	139	5070	142	223.7	381.3	3115.4	8349.1	0.02	0.61	0.373
	132	AO-60%RO-5	AO-RO	127	2.75	3.08	3.45	163	44.3	68.65	136	4966	139	223.7	374.1	3179.5	8308.4	0.02	0.6	0.383
	133	AO-60%RO-6	AO-RO	131	2.75	3.08	3.38	162	46.02	71.32	139	4865	142	228	388.6	3246.3	8273.3	0.02	0.59	0.392
	134	AO-60%RO-7	AO-RO	123	2.91	3.2	3.58	153	43.46	68.65	139	5037	142	219.4	381.3	3066.6	8256.5	0.019	0.61	0.371
	135	AO-80%RO-1	AO-RO	117	2.69	3.02	3.38	172	40.27	66.09	136	5309	139	215.3	367	2913.3	8394	0.021	0.63	0.347
	136	AO-80%RO-2	AO-RO	127	2.75	3.08	3.45	176	44.3	69.97	139	4993	142	223.7	381.3	3145.6	8314.8	0.021	0.6	0.378
	137	AO-80%RO-3	AO-RO	121	2.85	3.2	3.58	178	41.84	68.65	139	5117	142	219.4	374.1	2931.8	8227.4	0.022	0.62	0.356
	138	AO-80%RO-4	AO-RO	126	2.8	3.14	3.52	183	43.46	69.97	139	4949	142	223.7	388.6	3062.2	8193.7	0.022	0.6	0.374
	139	AO-80%RO-5	AO-RO	126	2.85	3.14	3.52	168	43.46	68.65	136	4935	139	223.7	381.3	3149.7	8252.6	0.02	0.6	0.382
	140	AO-80%RO-6	AO-RO	132	2.85	3.14	3.52	166	45.15	71.32	139	4825	142	228	388.6	3231.2	8222.5	0.02	0.59	0.393
	141	AO-80%RO-7	AO-RO	132	2.85	3.2	3.52	157	45.15	71.32	139	4819	142	228	388.6	3218.8	8195.8	0.019	0.59	0.393
	1101	AO-16	AO	114	2.91	3.2	3.52	143	41.05	64.84	134	5288	136	211.2	353.3	2873.1	8304	0.017	0.64	0.346
Validation	1102	AO-17	AO	119	2.91	3.2	3.58	142	43.46	67.36	136	5159	139	215.3	367	2960.1	8260.8	0.017	0.62	0.358
	1103	AO-18	AO	132	2.8	3.08	3.45	149	47.81	71.32	139	4845	142	228	381.3	3235.9	8229.9	0.018	0.59	0.393

1104	AO-19	AO	132	2.8	3.14	3.45	144	47.81	71.32	139	4820	142	228	381.3	3241.1	8205.7	0.018	0.59	0.395
1105	AO-20	AO	128	2.85	3.2	3.52	142	46.02	69.97	136	4887	139	223.7	374.1	3155.2	8184	0.017	0.6	0.386
1106	AO-10%SO-8	AO-SO	118	1.99	2.27	2.54	192	43.46	66.09	131	5267	134	211.2	353.3	3209.3	8668.7	0.022	0.61	0.37
1107	AO-10%SO-9	AO-SO	121	1.91	2.14	2.4	211	45.15	66.09	129	5224	131	215.3	346.6	3348.9	8784.5	0.024	0.6	0.381
1108	AO-10%SO-10	AO-SO	118	1.91	2.14	2.4	205	45.15	64.84	129	5289	131	211.2	346.6	3316.3	8809.8	0.023	0.6	0.376
1109	AO-20%SO-8	AO-SO	121	1.84	2.06	2.31	198	45.15	64.84	126	5337	129	215.3	346.6	3383	8918.7	0.022	0.6	0.379
1110	AO-20%SO-9	AO-SO	124	1.84	2.06	2.31	208	46.9	66.09	129	5261	131	219.4	353.3	3421.2	8889.6	0.023	0.59	0.385
1111	AO-20%SO-10	AO-SO	124	1.84	2.06	2.27	204	46.9	66.09	129	5263	131	219.4	353.3	3423.1	8890.4	0.023	0.59	0.385
1112	AO-30%SO-8	AO-SO	121	1.95	2.18	2.45	190	43.46	66.09	131	5262	134	215.3	360.1	3286.6	8739.1	0.022	0.6	0.376
1113	AO-30%SO-9	AO-SO	124	1.95	2.18	2.5	218	45.15	66.09	131	5138	134	219.4	360.1	3363.8	8719.9	0.025	0.59	0.386
1114	AO-30%SO-10	AO-SO	120	1.99	2.27	2.54	208	43.46	64.84	129	5250	131	215.3	353.3	3286.8	8745.2	0.024	0.6	0.376
1115	AO-40%SO-8	AO-SO	128	1.99	2.23	2.5	207	46.02	67.36	131	5044	134	223.7	374.1	3454.1	8704.5	0.024	0.58	0.397
1116	AO-40%SO-9	AO-SO	123	1.88	2.1	2.31	206	45.15	64.84	129	5224	131	219.4	360.1	3405.1	8835	0.023	0.59	0.385
1117	AO-40%SO-10	AO-SO	129	1.84	2.06	2.31	216	46.9	67.36	131	5117	134	228	367	3476.6	8808.9	0.024	0.58	0.395
1118	AO-60%SO-8	AO-SO	134	1.99	2.23	2.54	214	47.81	68.65	134	4908	136	232.4	388.6	3543.8	8666.1	0.025	0.57	0.409
1119	AO-60%SO-9	AO-SO	129	1.99	2.23	2.54	212	45.15	67.36	134	5061	136	228	381.3	3422.1	8695.2	0.024	0.58	0.394
1120	AO-60%SO-10	AO-SO	137	1.99	2.23	2.5	221	48.72	69.97	136	4884	139	236.8	396.1	3557.4	8662.7	0.026	0.56	0.411
1121	AO-80%SO-8	AO-SO	140	1.91	2.14	2.4	215	50.62	69.97	136	4849	139	241.4	396.1	3660.9	8724.5	0.025	0.56	0.42
1122	AO-80%SO-9	AO-SO	139	1.95	2.18	2.45	220	49.66	69.97	136	4901	139	241.4	396.1	3608	8729.7	0.025	0.56	0.413
1123	AO-80%SO-10	AO-SO	135	1.99	2.23	2.5	205	46.9	67.36	134	5001	136	236.8	388.6	3548.1	8754.8	0.023	0.57	0.405
1124	AO-10%CO-8	AO-CO	123	1.99	2.23	2.5	198	46.02	67.36	131	5253	134	219.4	353.3	3324.1	8775.4	0.023	0.6	0.379

1125	AO-10%CO-9	AO-CO	120	2.23	2.5	2.75	187	44.3	66.09	131	5250	134	215.3	353.3	3208.9	8645.2	0.022	0.61	0.371
1126	AO-10%CO-10	AO-CO	115	2.14	2.36	2.64	178	42.64	63.62	129	5433	131	211.2	340.1	3148.1	8759.3	0.02	0.62	0.359
1127	AO-20%CO-8	AO-CO	118	2.1	2.36	2.64	197	42.64	64.84	131	5330	134	215.3	353.3	3187.9	8715.3	0.023	0.61	0.366
1128	AO-20%CO-9	AO-CO	118	2.31	2.59	2.91	179	42.64	64.84	131	5290	134	215.3	353.3	3145.1	8614.2	0.021	0.61	0.365
1129	AO-20%CO-10	AO-CO	122	2.18	2.45	2.69	199	44.3	66.09	131	5237	134	219.4	353.3	3228.3	8664.4	0.023	0.6	0.373
1130	AO-30%CO-8	AO-CO	119	2.23	2.5	2.75	193	43.46	64.84	131	5297	134	215.3	353.3	3175.2	8665.5	0.022	0.61	0.366
1131	AO-30%CO-9	AO-CO	125	2.31	2.59	2.91	188	45.15	67.36	134	5121	136	223.7	367	3254.2	8563.7	0.022	0.6	0.38
1132	AO-30%CO-10	AO-CO	124	2.23	2.5	2.8	199	45.15	66.09	134	5194	136	223.7	367	3235.8	8629.6	0.023	0.6	0.375
1133	AO-40%CO-8	AO-CO	132	2.02	2.27	2.5	208	47.81	68.65	134	5049	136	232.4	374.1	3481.5	8737.9	0.024	0.58	0.398
1134	AO-40%CO-9	AO-CO	122	2.23	2.5	2.75	190	43.46	64.84	131	5223	134	219.4	360.1	3268.5	8681	0.022	0.6	0.377
1135	AO-40%CO-10	AO-CO	124	2.27	2.5	2.8	180	44.3	66.09	134	5205	136	223.7	367	3243.9	8628.8	0.021	0.6	0.376
1136	AO-60%CO-8	AO-CO	128	2.18	2.45	2.75	199	44.3	67.36	134	5093	136	228	381.3	3372.3	8664.3	0.023	0.59	0.389
1137	AO-60%CO-9	AO-CO	137	2.36	2.64	2.91	193	46.9	71.32	139	4860	142	236.8	396.1	3434	8487	0.023	0.57	0.405
1138	AO-60%CO-10	AO-CO	131	2.31	2.59	2.91	200	45.15	68.65	136	5025	139	232.4	381.3	3333.6	8559	0.023	0.59	0.389
1139	AO-80%CO-8	AO-CO	135	2.54	2.85	3.2	183	44.3	68.65	136	4830	139	236.8	396.1	3365	8378.1	0.022	0.58	0.402
1140	AO-80%CO-9	AO-CO	132	2.18	2.45	2.75	203	44.3	68.65	136	5013	139	232.4	388.6	3400	8616.3	0.024	0.58	0.395
1141	AO-80%CO-10	AO-CO	131	2.5	2.8	3.14	188	42.64	68.65	136	4974	139	232.4	388.6	3291.3	8453.5	0.022	0.59	0.389
1142	AO-10%RO-8	AO-RO	116	2.69	3.02	3.38	169	41.84	66.09	134	5272	136	211.2	353.3	2945	8385.2	0.02	0.63	0.351
1143	AO-10%RO-9	AO-RO	122	2.69	2.96	3.26	157	45.15	67.36	134	5042	136	215.3	360.1	3152.2	8350.8	0.019	0.6	0.377
1144	AO-10%RO-10	AO-RO	125	2.5	2.8	3.08	170	45.15	68.65	134	5082	136	219.4	367	3211.3	8463.4	0.02	0.6	0.379
1145	AO-20%RO-8	AO-RO	124	2.5	2.75	3.02	172	45.15	68.65	134	5086	136	219.4	367	3203.3	8461.7	0.02	0.6	0.379

1146	AO-20%RO-9	AO-RO	117	2.69	2.96	3.32	150	42.64	66.09	134	5195	136	211.2	360.1	3023.4	8368.8	0.018	0.62	0.361
1147	AO-20%RO-10	AO-RO	123	2.54	2.85	3.14	164	44.3	67.36	134	5101	136	219.4	360.1	3148	8413.1	0.019	0.61	0.374
1148	AO-30%RO-8	AO-RO	126	2.5	2.8	3.08	171	45.15	68.65	136	5107	139	223.7	367	3173.7	8452.2	0.02	0.6	0.375
1149	AO-30%RO-9	AO-RO	119	2.54	2.8	3.14	171	43.46	66.09	134	5223	136	215.3	360.1	3097	8491.4	0.02	0.62	0.365
1150	AO-30%RO-10	AO-RO	124	2.69	2.96	3.32	162	44.3	67.36	134	5062	136	219.4	367	3173.5	8397.6	0.019	0.6	0.378
1151	AO-40%RO-8	AO-RO	126	2.54	2.8	3.14	175	45.15	68.65	136	5028	139	223.7	374.1	3207.6	8411.1	0.021	0.6	0.381
1152	AO-40%RO-9	AO-RO	124	2.64	2.91	3.26	160	44.3	68.65	136	5032	139	219.4	374.1	3143.3	8335.8	0.019	0.6	0.377
1153	AO-40%RO-10	AO-RO	120	2.8	3.08	3.45	156	42.64	67.36	134	5147	136	215.3	360.1	3067.3	8370.2	0.019	0.62	0.366
1154	AO-60%RO-8	AO-RO	127	2.59	2.91	3.26	177	45.15	68.65	136	4992	139	223.7	381.3	3212.4	8380.9	0.021	0.6	0.383
1155	AO-60%RO-9	AO-RO	120	2.91	3.26	3.65	148	42.64	67.36	136	5132	139	215.3	367	3008.8	8288.2	0.018	0.62	0.363
1156	AO-60%RO-10	AO-RO	120	2.8	3.14	3.45	158	42.64	67.36	136	5145	139	215.3	367	3040.4	8343.1	0.019	0.62	0.364
1157	AO-80%RO-8	AO-RO	121	2.69	3.02	3.38	177	42.64	67.36	139	5179	142	219.4	374.1	2999.5	8355.7	0.021	0.62	0.359
1158	AO-80%RO-9	AO-RO	127	2.85	3.2	3.58	157	44.3	69.97	139	4972	142	223.7	381.3	3102.7	8231.8	0.019	0.6	0.377
1159	AO-80%RO-10	AO-RO	121	2.85	3.2	3.58	160	41.84	67.36	136	5106	139	219.4	374.1	3009.5	8276	0.019	0.62	0.364

Table S5. Data used for the establishment of PLSR and SVR models.

Cal/Val	Number	Level	Group	T2W	T21S	T21P	T21E	S21	T22S	T22P	T22E	S22	T23S	T23P	T23E	S23	STotal	P21	P22	P23
Calibration	1	0	AO	114.7	2.8	3.1	3.5	140.8	41.8	64.8	133.7	5316.8	136.3	211.2	353.3	2905.5	8363.1	0.02	0.64	0.35
	2	0	AO	120.5	2.8	3.1	3.5	138.1	43.5	67.4	133.7	5135.7	136.3	215.3	360.1	3046.8	8320.6	0.02	0.62	0.37
	3	0	AO	126.2	2.69	3	3.3	145.6	45.1	68.7	136.3	5006.8	138.92	223.7	374.1	3140.8	8293.1	0.02	0.6	0.38
	4	0	AO	119.4	2.85	3.1	3.5	148.6	43.5	66.1	133.7	5102	136.3	215.3	360.1	3016.7	8267.3	0.02	0.62	0.37
	5	0	AO	124.2	2.85	3.1	3.5	147.7	45.1	68.7	136.3	4989.1	138.92	219.4	374.1	3097.2	8233.9	0.02	0.61	0.38
	6	0	AO	114.7	2.91	3.2	3.6	140.3	41.8	64.8	133.7	5271.1	136.3	211.2	353.3	2884.5	8295.9	0.02	0.64	0.35
	7	0	AO	126.1	2.8	3.1	3.5	144.9	45.1	68.7	136.3	4975.9	138.92	223.7	374.1	3112.4	8233.3	0.02	0.6	0.38
	8	0	AO	123.6	2.85	3.1	3.5	140	44.3	67.4	133.7	5015.3	136.3	219.4	367	3122.3	8277.5	0.02	0.61	0.38
	9	0	AO	128	2.75	3.1	3.4	142.4	46	70	136.3	4934	138.92	223.7	374.1	3178.6	8254.9	0.02	0.6	0.39
	10	0	AO	123.3	2.8	3.1	3.5	142.9	44.3	68.7	136.3	5063.5	138.92	219.4	367	3054.6	8261	0.02	0.61	0.37
	11	0	AO	128	2.75	3.1	3.4	146	46	70	136.3	4915	138.92	223.7	374.1	3173.6	8234.5	0.02	0.6	0.39
	12	0	AO	123.3	2.91	3.3	3.7	141.8	44.3	68.7	136.3	5037.1	138.92	219.4	367	3035.8	8214.7	0.02	0.61	0.37
	13	0	AO	117.5	2.85	3.1	3.5	132.9	42.6	66.1	133.7	5167.6	136.3	211.2	360.1	2999.8	8300.2	0.02	0.62	0.36
	14	0	AO	124.6	2.8	3.1	3.4	140.7	45.1	68.7	133.7	4998.2	136.3	219.4	360.1	3131	8269.9	0.02	0.6	0.38
	15	0	AO	128.7	2.8	3.1	3.5	143.6	46.9	70	136.3	4886.8	138.92	223.7	374.1	3210.7	8241.1	0.02	0.59	0.39
	16	0.1	AO-SO	112.3	2.06	2.4	2.7	174.3	41	64.8	131.2	5488	133.73	203.3	346.6	3071.1	8733.3	0.02	0.63	0.35

17	0.1	AO-SO	115	2.06	2.4	2.7	183.4	41.8	64.8	131.2	5371.1	133.73	207.3	353.3	3143.9	8698.5	0.02	0.62	0.36
18	0.1	AO-SO	114.1	1.91	2.1	2.4	199.3	43.5	63.6	128.7	5440.7	131.21	207.3	340.1	3192.2	8832.3	0.02	0.62	0.36
19	0.1	AO-SO	115.6	1.91	2.2	2.4	199.7	43.5	64.8	128.7	5384	131.21	207.3	346.6	3225.6	8809.3	0.02	0.61	0.37
20	0.1	AO-SO	124.6	1.95	2.2	2.4	216.4	46.9	67.4	131.2	5152.9	133.73	219.4	360.1	3391	8760.2	0.03	0.59	0.39
21	0.1	AO-SO	114	1.91	2.1	2.4	194.6	43.5	63.6	128.7	5470.8	131.21	207.3	340.1	3190.7	8856.2	0.02	0.62	0.36
22	0.1	AO-SO	120.8	1.88	2.1	2.4	209.2	45.1	66.1	128.7	5296.3	131.21	215.3	346.6	3326.3	8831.8	0.02	0.6	0.38
23	0.2	AO-SO	115.4	1.95	2.2	2.4	175.1	42.6	63.6	131.2	5486.5	133.73	211.2	353.3	3170.1	8831.7	0.02	0.62	0.36
24	0.2	AO-SO	120.2	1.99	2.2	2.5	188.5	44.3	64.8	128.7	5302.3	131.21	215.3	353.3	3323.6	8814.4	0.02	0.6	0.38
25	0.2	AO-SO	121.5	1.91	2.1	2.4	190.2	45.1	66.1	131.2	5256.7	133.73	215.3	353.3	3344.2	8791.2	0.02	0.6	0.38
26	0.2	AO-SO	120.3	1.84	2.1	2.3	199.8	45.1	64.8	126.3	5344.5	128.73	215.3	340.1	3367.8	8912.1	0.02	0.6	0.38

27	0.2	AO-SO	127.9	1.88	2.1	2.4	210.1	48.7	68.7	131.2	5177.2	133.73	223.7	360.1	3475.3	8862.6	0.02	0.58	0.39
28	0.2	AO-SO	119.8	1.88	2.1	2.4	194.8	45.1	64.8	126.3	5392.5	128.73	215.3	340.1	3340.1	8927.3	0.02	0.6	0.37
29	0.2	AO-SO	122.7	1.88	2.1	2.3	192.6	46	66.1	131.2	5337.4	133.73	219.4	353.3	3360	8890	0.02	0.6	0.38
30	0.3	AO-SO	119.4	2.02	2.3	2.5	192.4	42.6	64.8	128.7	5307.9	131.21	215.3	353.3	3258.2	8758.5	0.02	0.61	0.37
31	0.3	AO-SO	124.7	1.99	2.2	2.5	197.1	45.1	67.4	131.2	5152.2	133.73	219.4	367	3372.3	8721.7	0.02	0.59	0.39
32	0.3	AO-SO	120.6	1.91	2.1	2.4	205.3	44.3	64.8	128.7	5253.3	131.21	215.3	353.3	3346.7	8805.3	0.02	0.6	0.38
33	0.3	AO-SO	123.9	1.88	2.1	2.4	213.7	46	66.1	131.2	5170.5	133.73	219.4	360.1	3400.7	8784.9	0.02	0.59	0.39
34	0.3	AO-SO	128.1	1.91	2.2	2.4	219.6	46.9	68.7	133.7	5045.2	136.3	223.7	367	3426.1	8690.8	0.03	0.58	0.39
35	0.3	AO-SO	118.9	1.91	2.1	2.4	194.6	43.5	64.8	131.2	5374.6	133.73	215.3	353.3	3247.3	8816.5	0.02	0.61	0.37
36	0.3	AO-SO	124.7	1.99	2.2	2.5	198.3	45.1	67.4	133.7	5281.4	136.3	223.7	367	3302.5	8782.2	0.02	0.6	0.38

37	0.4	AO-SO	122.2	1.95	2.2	2.5	200.1	43.5	64.8	128.7	5220.6	131.21	219.4	360.1	3329.9	8750.6	0.02	0.6	0.38
38	0.4	AO-SO	125.3	1.99	2.2	2.5	204	45.1	66.1	131.2	5160.5	133.73	223.7	367	3362.9	8727.4	0.02	0.59	0.39
39	0.4	AO-SO	124.4	1.95	2.2	2.4	204.2	45.1	66.1	128.7	5155.1	131.21	219.4	360.1	3423.5	8782.8	0.02	0.59	0.39
40	0.4	AO-SO	127.7	1.84	2.1	2.4	209.9	46	67.4	131.2	5082.1	133.73	223.7	367	3468.2	8760.2	0.02	0.58	0.4
41	0.4	AO-SO	133.8	1.88	2.1	2.4	218.5	48.7	70	133.7	5016.6	136.3	232.4	374.1	3540.4	8775.4	0.03	0.57	0.4
42	0.4	AO-SO	122.6	2.02	2.3	2.5	192.3	44.3	66.1	131.2	5263.6	133.73	219.4	367	3306.3	8762.2	0.02	0.6	0.38
43	0.4	AO-SO	127.6	1.99	2.2	2.5	205.1	46	67.4	133.7	5175.5	136.3	228	374.1	3362.3	8742.9	0.02	0.59	0.39
44	0.6	AO-SO	126	1.99	2.2	2.5	202	44.3	66.1	131.2	5121.3	133.73	223.7	374.1	3397.2	8720.5	0.02	0.59	0.39
45	0.6	AO-SO	129.1	1.99	2.3	2.5	211.3	46	67.4	133.7	5057.4	136.3	228	381.3	3432.1	8700.9	0.02	0.58	0.39
46	0.6	AO-SO	133.8	1.99	2.3	2.5	210.1	47.8	68.7	133.7	4931.7	136.3	232.4	381.3	3536.2	8678	0.02	0.57	0.41

47	0.6	AO-SO	128.4	1.99	2.2	2.5	211.3	45.1	66.1	131.2	5071.2	133.73	228	374.1	3438.1	8720.6	0.02	0.58	0.39
48	0.6	AO-SO	133	1.99	2.2	2.5	215.3	46.9	68.7	133.7	4969.6	136.3	232.4	381.3	3501.2	8686.1	0.03	0.57	0.4
49	0.6	AO-SO	128.6	1.99	2.2	2.5	201.2	46	67.4	133.7	5129.9	136.3	228	381.3	3418.6	8749.7	0.02	0.59	0.39
50	0.6	AO-SO	133.7	1.95	2.2	2.4	210.9	47.8	68.7	136.3	5054.3	138.92	236.8	388.6	3454.5	8719.7	0.02	0.58	0.4
51	0.8	AO-SO	132.9	1.91	2.1	2.4	206.6	46.9	67.4	131.2	4994.3	133.73	232.4	381.3	3565.4	8766.2	0.02	0.57	0.41
52	0.8	AO-SO	136.6	1.91	2.1	2.4	213	47.8	68.7	133.7	4916.1	136.3	236.8	388.6	3614.4	8743.5	0.02	0.56	0.41
53	0.8	AO-SO	136	1.91	2.1	2.4	208.6	46.9	68.7	133.7	4958.5	136.3	236.8	388.6	3585.7	8752.8	0.02	0.57	0.41
54	0.8	AO-SO	139.6	1.91	2.1	2.4	218.4	49.7	70	136.3	4877.3	138.92	241.4	396.1	3634.6	8730.2	0.03	0.56	0.42
55	0.8	AO-SO	144.4	1.95	2.2	2.4	216.8	50.6	72.7	138.9	4789.1	141.6	246	411.5	3684.8	8690.6	0.03	0.55	0.42
56	0.8	AO-SO	134.4	1.99	2.2	2.5	200.9	46.9	68.7	136.3	5039.2	138.92	236.8	388.6	3496.5	8736.7	0.02	0.58	0.4

57	0.8	AO-SO	139.4	2.02	2.3	2.5	203.8	48.7	70	136.3	4894.5	138.92	241.4	396.1	3606.1	8704.3	0.02	0.56	0.41
58	###	SO	141.3	2.96	3.3	3.7	147.6	46	70	138.9	4592.9	141.6	246	411.5	3326.2	8066.7	0.02	0.57	0.41
59	###	SO	151.6	2.85	3.2	3.5	155.2	50.6	74.1	144.3	4364.3	147.09	255.6	435.7	3475.9	7995.5	0.02	0.55	0.44
60	###	SO	147.1	3.02	3.3	3.7	145.8	48.7	72.7	141.6	4465.9	144.32	250.8	419.4	3408.3	8020	0.02	0.56	0.43
61	###	SO	151.6	2.96	3.3	3.7	144.9	49.7	74.1	144.3	4367.3	147.09	255.6	435.7	3465.6	7977.8	0.02	0.55	0.43
62	0.1	AO-CO	119.8	2.1	2.3	2.6	189.6	44.3	66.1	131.2	5315.1	133.73	215.3	346.6	3226.8	8731.5	0.02	0.61	0.37
63	0.1	AO-CO	117.6	2.02	2.2	2.5	192.8	43.5	64.8	128.7	5361.2	131.21	211.2	346.6	3253.7	8807.8	0.02	0.61	0.37
64	0.1	AO-CO	119.8	2.1	2.3	2.6	189.6	44.3	66.1	131.2	5315.1	133.73	215.3	346.6	3226.8	8731.5	0.02	0.61	0.37
65	0.1	AO-CO	116.7	2.23	2.4	2.7	180.9	43.5	64.8	128.7	5341.3	131.21	211.2	346.6	3151.9	8674.1	0.02	0.62	0.36
66	0.1	AO-CO	123.7	2.23	2.4	2.7	186.7	46	67.4	133.7	5158.9	136.3	219.4	360.1	3268	8613.7	0.02	0.6	0.38
67	0.1	AO-CO	116	2.4	2.7	3	176	42.6	64.8	131.2	5333	133.73	211.2	346.6	3071.5	8580.5	0.02	0.62	0.36
68	0.1	AO-CO	121.7	2.4	2.6	3	182.1	45.1	67.4	133.7	5231.3	136.3	219.4	353.3	3133.4	8546.8	0.02	0.61	0.37

69	0.2	AO-CO	112.3	2.1	2.4	2.6	191.5	41	63.6	131.2	5511.6	133.73	207.3	346.6	3045	8748.1	0.02	0.63	0.35
70	0.2	AO-CO	123.1	2.1	2.4	2.6	201.7	45.1	67.4	133.7	5212.2	136.3	219.4	367	3271.1	8684.9	0.02	0.6	0.38
71	0.2	AO-CO	116.1	2.18	2.4	2.7	191.9	42.6	63.6	128.7	5333.4	131.21	211.2	346.6	3168.4	8693.6	0.02	0.61	0.36
72	0.2	AO-CO	122.7	2.31	2.6	2.9	188.4	45.1	67.4	133.7	5196.6	136.3	219.4	360.1	3205.3	8590.2	0.02	0.61	0.37
73	0.2	AO-CO	126.3	2.31	2.5	2.9	191.7	46	68.7	136.3	5096.4	138.92	223.7	367	3261.9	8550	0.02	0.6	0.38
74	0.2	AO-CO	119.5	2.31	2.5	2.9	167.7	43.5	66.1	131.2	5285.1	133.73	215.3	353.3	3150.1	8602.9	0.02	0.61	0.37
75	0.2	AO-CO	123.2	2.31	2.5	2.9	176.5	45.1	67.4	133.7	5170.2	136.3	219.4	360.1	3224	8570.8	0.02	0.6	0.38
76	0.3	AO-CO	116.9	2.14	2.4	2.7	192.1	41.8	63.6	131.2	5392	133.73	215.3	353.3	3144.1	8728.2	0.02	0.62	0.36
77	0.3	AO-CO	122.4	2.1	2.4	2.6	202.7	44.3	66.1	131.2	5220.1	133.73	219.4	360.1	3278.4	8701.2	0.02	0.6	0.38
78	0.3	AO-CO	126.2	2.1	2.4	2.6	210.5	46	67.4	133.7	5111.1	136.3	223.7	367	3350.9	8672.5	0.02	0.59	0.39

79	0.3	AO-CO	120.6	2.31	2.6	2.9	179.6	42.6	66.1	133.7	5284.9	136.3	219.4	360.1	3128.9	8593.4	0.02	0.62	0.36
80	0.3	AO-CO	129.1	2.31	2.6	2.9	189.2	46	68.7	136.3	5020.1	138.92	228	374.1	3313.4	8522.7	0.02	0.59	0.39
81	0.3	AO-CO	121.3	2.45	2.7	3	175.9	43.5	66.1	133.7	5199.2	136.3	219.4	360.1	3140.4	8515.4	0.02	0.61	0.37
82	0.3	AO-CO	125.6	2.4	2.7	3	184.7	45.1	68.7	136.3	5104.1	138.92	223.7	374.1	3196.1	8484.9	0.02	0.6	0.38
83	0.4	AO-CO	121.8	2.02	2.3	2.5	199.5	44.3	64.8	128.7	5285.7	131.21	219.4	353.3	3326.2	8811.4	0.02	0.6	0.38
84	0.4	AO-CO	128.4	2.06	2.3	2.5	201	46	67.4	131.2	5152.7	133.73	228	367	3414.7	8768.3	0.02	0.59	0.39
85	0.4	AO-CO	127.7	2.27	2.5	2.8	194.8	45.1	67.4	133.7	5124.2	136.3	228	374.1	3327.6	8646.6	0.02	0.59	0.39
86	0.4	AO-CO	128.7	2.27	2.5	2.9	181	45.1	68.7	136.3	5098.4	138.92	228	374.1	3312.8	8592.2	0.02	0.59	0.39
87	0.4	AO-CO	133.6	2.27	2.5	2.8	189.4	47.8	70	136.3	4940.5	138.92	232.4	388.6	3430	8559.8	0.02	0.58	0.4
88	0.4	AO-CO	125.2	2.5	2.7	3.1	174.7	44.3	67.4	133.7	5132.4	136.3	223.7	367	3228.9	8536.1	0.02	0.6	0.38

89	0.4	AO-CO	128.9	2.45	2.7	3	179.7	46	68.7	136.3	5030.2	138.92	228	381.3	3292	8501.9	0.02	0.59	0.39
90	0.6	AO-CO	122.5	2.14	2.4	2.7	197.9	42.6	64.8	133.7	5275.3	136.3	223.7	367	3237.2	8710.5	0.02	0.61	0.37
91	0.6	AO-CO	135.3	2.18	2.4	2.7	203.2	46.9	70	136.3	4963.2	138.92	236.8	388.6	3458.7	8625.1	0.02	0.58	0.4
92	0.6	AO-CO	126.5	2.31	2.6	2.9	198.3	43.5	66.1	133.7	5108.7	136.3	228	374.1	3282.2	8589.1	0.02	0.6	0.38
93	0.6	AO-CO	127.7	2.4	2.6	3	180.7	43.5	67.4	133.7	5088.5	136.3	228	374.1	3287.1	8556.2	0.02	0.6	0.38
94	0.6	AO-CO	131.7	2.36	2.6	3	189.6	45.1	68.7	136.3	4975.7	138.92	232.4	388.6	3359.9	8525.3	0.02	0.58	0.39
95	0.6	AO-CO	130.8	2.54	2.9	3.2	182.8	44.3	68.7	136.3	4982.4	138.92	232.4	388.6	3277.1	8442.4	0.02	0.59	0.39
96	0.6	AO-CO	134.8	2.5	2.8	3.1	184	45.1	70	138.9	4886.2	141.6	236.8	396.1	3343.9	8414.1	0.02	0.58	0.4
97	0.8	AO-CO	127.1	2.18	2.4	2.7	196.5	42.6	66.1	133.7	5119.7	136.3	228	381.3	3337.9	8654.1	0.02	0.59	0.39
98	0.8	AO-CO	137.1	2.23	2.5	2.8	207.7	46	70	136.3	4836	138.92	236.8	403.7	3532.5	8576.2	0.02	0.56	0.41

99	0.8	AO-CO	131.2	2.36	2.6	3	195.6	43.5	68.7	136.3	5007.3	138.92	232.4	388.6	3346.1	8548.9	0.02	0.59	0.39
100	0.8	AO-CO	136.2	2.36	2.6	3	202.4	45.1	70	136.3	4850.2	138.92	236.8	396.1	3463.6	8516.2	0.02	0.57	0.41
101	0.8	AO-CO	136.5	2.5	2.8	3.1	191.8	45.1	70	136.3	4789.8	138.92	236.8	396.1	3429.2	8410.8	0.02	0.57	0.41
102	0.8	AO-CO	140.7	2.54	2.9	3.2	194.5	46.9	71.3	138.9	4689.2	141.6	241.4	403.7	3496.1	8379.8	0.02	0.56	0.42
103	0.8	AO-CO	139.7	2.54	2.9	3.2	186.4	46	71.3	138.9	4726.9	141.6	241.4	403.7	3430.4	8343.8	0.02	0.57	0.41
104	###	CO	136.3	3.08	3.5	3.9	152.4	42.6	68.7	136.3	4642.9	138.92	236.8	403.7	3324.7	8120	0.02	0.57	0.41
105	###	CO	147.7	2.91	3.3	3.7	159	46.9	72.7	138.9	4342.1	141.6	246	419.4	3547.4	8048.5	0.02	0.54	0.44
106	###	CO	144.5	2.96	3.3	3.8	153.7	45.1	71.3	138.9	4473.3	141.6	246	411.5	3439.9	8066.8	0.02	0.56	0.43
107	###	CO	147.9	3.08	3.5	3.8	157.3	46.9	72.7	138.9	4326.6	141.6	246	419.4	3548.9	8032.8	0.02	0.54	0.44
108	0.1	AO-RO	117.1	2.75	3	3.4	151.7	42.6	66.1	133.7	5219.5	136.3	211.2	353.3	3013.6	8384.7	0.02	0.62	0.36
109	0.1	AO-RO	118.7	2.5	2.8	3.1	166.2	43.5	66.1	133.7	5266.2	136.3	215.3	360.1	3064.8	8497.2	0.02	0.62	0.36
110	0.1	AO-RO	120.7	2.69	3	3.3	172.8	43.5	67.4	133.7	5093.7	136.3	215.3	360.1	3086.8	8353.3	0.02	0.61	0.37

111	0.1	AO-RO	117.1	2.75	3	3.4	151.7	42.6	66.1	133.7	5219.5	136.3	211.2	353.3	3013.6	8384.7	0.02	0.62	0.36
112	0.1	AO-RO	128.4	2.64	2.9	3.3	157	46.9	70	136.3	4933.5	138.92	223.7	367	3232.8	8323.2	0.02	0.59	0.39
113	0.1	AO-RO	117.3	2.69	3	3.3	151.1	43.5	66.1	133.7	5192.4	136.3	211.2	353.3	3018.3	8361.8	0.02	0.62	0.36
114	0.1	AO-RO	123.6	2.75	3	3.3	154.1	45.1	67.4	133.7	5030	136.3	219.4	360.1	3143.8	8328	0.02	0.6	0.38
115	0.2	AO-RO	114.8	2.5	2.8	3.1	163.8	41	64.8	131.2	5387.9	133.73	211.2	346.6	2981.3	8533	0.02	0.63	0.35
116	0.2	AO-RO	118.7	2.45	2.7	3	170.8	43.5	66.1	133.7	5261.4	136.3	215.3	360.1	3067.5	8499.7	0.02	0.62	0.36
117	0.2	AO-RO	116.7	2.54	2.8	3.1	160.5	42.6	66.1	133.7	5274.2	136.3	211.2	353.3	3013	8447.7	0.02	0.62	0.36
118	0.2	AO-RO	123.8	2.64	3	3.3	159.7	45.1	67.4	133.7	5016.6	136.3	219.4	367	3163.3	8339.6	0.02	0.6	0.38
119	0.2	AO-RO	128.6	2.59	2.9	3.2	162.1	46	70	136.3	4908.2	138.92	223.7	374.1	3240.8	8311.1	0.02	0.59	0.39
120	0.2	AO-RO	118.7	2.85	3.2	3.5	143.4	42.6	67.4	133.7	5119.3	136.3	211.2	360.1	3015.2	8278	0.02	0.62	0.36

121	0.2	AO-RO	123.8	2.85	3.1	3.5	151.3	44.3	68.7	136.3	5018.9	138.92	219.4	367	3081.2	8251.4	0.02	0.61	0.37
122	0.3	AO-RO	115.3	2.5	2.8	3.1	162.8	41	64.8	131.2	5356.6	133.73	211.2	353.3	3009.5	8528.8	0.02	0.63	0.35
123	0.3	AO-RO	125.5	2.5	2.8	3.1	171.3	45.1	68.7	136.3	5107.2	138.92	223.7	367	3173.7	8452.2	0.02	0.6	0.38
124	0.3	AO-RO	118.5	2.64	3	3.3	159.2	42.6	66.1	133.7	5243.4	136.3	215.3	360.1	3031.9	8434.5	0.02	0.62	0.36
125	0.3	AO-RO	118.5	2.64	3	3.3	159.2	42.6	66.1	133.7	5243.4	136.3	215.3	360.1	3031.9	8434.5	0.02	0.62	0.36
126	0.3	AO-RO	123.6	2.69	3	3.3	161.8	44.3	67.4	133.7	5062.3	136.3	219.4	367	3173.5	8397.6	0.02	0.6	0.38
127	0.3	AO-RO	116.6	2.8	3.1	3.5	150	41	66.1	133.7	5221.1	136.3	211.2	360.1	2969.8	8340.8	0.02	0.63	0.36
128	0.3	AO-RO	124.5	2.85	3.1	3.5	152	44.3	68.7	136.3	5007.1	138.92	219.4	374.1	3138.6	8297.7	0.02	0.6	0.38
129	0.4	AO-RO	115.8	2.59	2.9	3.3	167.5	41	66.1	133.7	5334.3	136.3	211.2	360.1	2975.9	8477.7	0.02	0.63	0.35
130	0.4	AO-RO	121.3	2.54	2.9	3.2	172.3	43.5	67.4	136.3	5203.5	138.92	219.4	367	3066.8	8442.6	0.02	0.62	0.36

131	0.4	AO-RO	119.1	2.69	3	3.3	158.8	42.6	66.1	133.7	5189.3	136.3	215.3	360.1	3055	8403.2	0.02	0.62	0.36
132	0.4	AO-RO	123.7	2.59	2.9	3.2	166.4	44.3	68.7	136.3	5072.5	138.92	219.4	374.1	3130	8368.9	0.02	0.61	0.37
133	0.4	AO-RO	129.3	2.69	3	3.3	161.3	46	70	136.3	4866.8	138.92	223.7	381.3	3278.3	8306.5	0.02	0.59	0.4
134	0.4	AO-RO	120	2.96	3.3	3.7	148.3	42.6	67.4	136.3	5142.6	138.92	215.3	367	3018.6	8309.5	0.02	0.62	0.36
135	0.4	AO-RO	124.9	2.85	3.2	3.5	151.7	44.3	68.7	136.3	4964.2	138.92	219.4	374.1	3147.7	8263.6	0.02	0.6	0.38
136	0.6	AO-RO	114.9	2.64	3	3.3	167.6	40.3	66.1	136.3	5366	138.92	211.2	367	2910.9	8444.6	0.02	0.64	0.35
137	0.6	AO-RO	121.5	2.59	2.9	3.2	170.9	43.5	67.4	136.3	5173.6	138.92	219.4	367	3069.7	8414.2	0.02	0.62	0.37
138	0.6	AO-RO	119.7	2.75	3.1	3.5	161.6	42.6	67.4	136.3	5182.3	138.92	215.3	367	3037	8380.9	0.02	0.62	0.36
139	0.6	AO-RO	125.2	2.64	3	3.3	163.7	44.3	68.7	138.9	5070	141.6	223.7	381.3	3115.4	8349.1	0.02	0.61	0.37
140	0.6	AO-RO	126.7	2.75	3.1	3.5	163.3	44.3	68.7	136.3	4965.6	138.92	223.7	374.1	3179.5	8308.4	0.02	0.6	0.38

141	0.6	AO-RO	131.5	2.75	3.1	3.4	162	46	71.3	138.9	4865	141.6	228	388.6	3246.3	8273.3	0.02	0.59	0.39
142	0.6	AO-RO	123.4	2.91	3.2	3.6	152.9	43.5	68.7	138.9	5037	141.6	219.4	381.3	3066.6	8256.5	0.02	0.61	0.37
143	0.8	AO-RO	116.6	2.69	3	3.4	172.2	40.3	66.1	136.3	5308.6	138.92	215.3	367	2913.3	8394	0.02	0.63	0.35
144	0.8	AO-RO	126.7	2.75	3.1	3.5	176	44.3	70	138.9	4993.1	141.6	223.7	381.3	3145.6	8314.8	0.02	0.6	0.38
145	0.8	AO-RO	121	2.85	3.2	3.6	178.2	41.8	68.7	138.9	5117.3	141.6	219.4	374.1	2931.8	8227.4	0.02	0.62	0.36
146	0.8	AO-RO	125.9	2.8	3.1	3.5	183	43.5	70	138.9	4948.5	141.6	223.7	388.6	3062.2	8193.7	0.02	0.6	0.37
147	0.8	AO-RO	126.5	2.85	3.1	3.5	167.9	43.5	68.7	136.3	4935	138.92	223.7	381.3	3149.7	8252.6	0.02	0.6	0.38
148	0.8	AO-RO	131.5	2.85	3.1	3.5	166.2	45.1	71.3	138.9	4825.1	141.6	228	388.6	3231.2	8222.5	0.02	0.59	0.39
149	0.8	AO-RO	131.5	2.85	3.2	3.5	157.5	45.1	71.3	138.9	4819.4	141.6	228	388.6	3218.8	8195.8	0.02	0.59	0.39
150	###	RO	119.4	3.02	3.4	3.8	149.8	40.3	67.4	138.9	5182.6	141.6	219.4	381.3	2866.1	8198.5	0.02	0.63	0.35
151	###	RO	124.9	3.08	3.5	3.9	149.2	42.6	68.7	138.9	4984.5	141.6	223.7	388.6	3020.6	8154.2	0.02	0.61	0.37
152	###	RO	125.8	3.02	3.4	3.7	145.6	43.5	70	141.6	4978.2	144.32	223.7	388.6	3024.5	8148.2	0.02	0.61	0.37

	153	###	RO	130.6	3.02	3.4	3.8	148.9	44.3	71.3	141.6	4829.7	144.32	228	396.1	3135.3	8114	0.02	0.6	0.39
	154	0	AO	114.4	2.91	3.2	3.5	143	41	64.8	133.7	5287.9	136.3	211.2	353.3	2873.1	8304	0.02	0.64	0.35
	155	0	AO	119.3	2.91	3.2	3.6	142.1	43.5	67.4	136.3	5158.6	138.92	215.3	367	2960.1	8260.8	0.02	0.62	0.36
	156	0	AO	131.7	2.8	3.1	3.5	149.1	47.8	71.3	138.9	4844.8	141.6	228	381.3	3235.9	8229.9	0.02	0.59	0.39
	157	0	AO	132	2.8	3.1	3.5	144.2	47.8	71.3	138.9	4820.4	141.6	228	381.3	3241.1	8205.7	0.02	0.59	0.4
	158	0	AO	128.1	2.85	3.2	3.5	141.9	46	70	136.3	4886.9	138.92	223.7	374.1	3155.2	8184	0.02	0.6	0.39
	159	0.1	AO-SO	118.4	1.99	2.3	2.5	192.3	43.5	66.1	131.2	5267.1	133.73	211.2	353.3	3209.3	8668.7	0.02	0.61	0.37
	160	0.1	AO-SO	121.4	1.91	2.1	2.4	211.4	45.1	66.1	128.7	5224.2	131.21	215.3	346.6	3348.9	8784.5	0.02	0.6	0.38
Validation	161	0.1	AO-SO	118.5	1.91	2.1	2.4	204.8	45.1	64.8	128.7	5288.7	131.21	211.2	346.6	3316.3	8809.8	0.02	0.6	0.38
	162	0.2	AO-SO	120.5	1.84	2.1	2.3	198.3	45.1	64.8	126.3	5337.4	128.73	215.3	346.6	3383	8918.7	0.02	0.6	0.38
	163	0.2	AO-SO	123.6	1.84	2.1	2.3	207.8	46.9	66.1	128.7	5260.6	131.21	219.4	353.3	3421.2	8889.6	0.02	0.59	0.39
	164	0.2	AO-SO	123.7	1.84	2.1	2.3	204.3	46.9	66.1	128.7	5263	131.21	219.4	353.3	3423.1	8890.4	0.02	0.59	0.39
	165	0.3	AO-SO	120.8	1.95	2.2	2.4	190.3	43.5	66.1	131.2	5262.2	133.73	215.3	360.1	3286.6	8739.1	0.02	0.6	0.38

166	0.3	AO-SO	123.7	1.95	2.2	2.5	217.8	45.1	66.1	131.2	5138.3	133.73	219.4	360.1	3363.8	8719.9	0.03	0.59	0.39
167	0.3	AO-SO	119.9	1.99	2.3	2.5	207.9	43.5	64.8	128.7	5250.4	131.21	215.3	353.3	3286.8	8745.2	0.02	0.6	0.38
168	0.4	AO-SO	127.8	1.99	2.2	2.5	206.7	46	67.4	131.2	5043.6	133.73	223.7	374.1	3454.1	8704.5	0.02	0.58	0.4
169	0.4	AO-SO	123	1.88	2.1	2.3	205.6	45.1	64.8	128.7	5224.4	131.21	219.4	360.1	3405.1	8835	0.02	0.59	0.39
170	0.4	AO-SO	129.1	1.84	2.1	2.3	215.6	46.9	67.4	131.2	5116.8	133.73	228	367	3476.6	8808.9	0.02	0.58	0.4
171	0.6	AO-SO	134	1.99	2.2	2.5	214.1	47.8	68.7	133.7	4908.1	136.3	232.4	388.6	3543.8	8666.1	0.03	0.57	0.41
172	0.6	AO-SO	129	1.99	2.2	2.5	211.8	45.1	67.4	133.7	5061.4	136.3	228	381.3	3422.1	8695.2	0.02	0.58	0.39
173	0.6	AO-SO	136.8	1.99	2.2	2.5	220.9	48.7	70	136.3	4884.4	138.92	236.8	396.1	3557.4	8662.7	0.03	0.56	0.41
174	0.8	AO-SO	140.2	1.91	2.1	2.4	215.1	50.6	70	136.3	4848.6	138.92	241.4	396.1	3660.9	8724.5	0.03	0.56	0.42
175	0.8	AO-SO	139.1	1.95	2.2	2.4	220.4	49.7	70	136.3	4901.4	138.92	241.4	396.1	3608	8729.7	0.03	0.56	0.41

176	0.8	AO-SO	134.5	1.99	2.2	2.5	205.3	46.9	67.4	133.7	5001.5	136.3	236.8	388.6	3548.1	8754.8	0.02	0.57	0.41
177	1	SO	146.8	2.85	3.2	3.6	151.3	47.8	72.7	141.6	4476.1	144.32	250.8	427.5	3401.5	8028.9	0.02	0.56	0.42
178	0.1	AO-CO	123.5	1.99	2.2	2.5	198.2	46	67.4	131.2	5253	133.73	219.4	353.3	3324.1	8775.4	0.02	0.6	0.38
179	0.1	AO-CO	120.1	2.23	2.5	2.7	186.7	44.3	66.1	131.2	5249.7	133.73	215.3	353.3	3208.9	8645.2	0.02	0.61	0.37
180	0.1	AO-CO	115.4	2.14	2.4	2.6	177.9	42.6	63.6	128.7	5433.3	131.21	211.2	340.1	3148.1	8759.3	0.02	0.62	0.36
181	0.2	AO-CO	118.5	2.1	2.4	2.6	196.9	42.6	64.8	131.2	5330.5	133.73	215.3	353.3	3187.9	8715.3	0.02	0.61	0.37
182	0.2	AO-CO	118.5	2.31	2.6	2.9	179.1	42.6	64.8	131.2	5290	133.73	215.3	353.3	3145.1	8614.2	0.02	0.61	0.37
183	0.2	AO-CO	121.8	2.18	2.4	2.7	198.5	44.3	66.1	131.2	5237.5	133.73	219.4	353.3	3228.3	8664.4	0.02	0.6	0.37
184	0.3	AO-CO	118.6	2.23	2.5	2.7	193.4	43.5	64.8	131.2	5296.9	133.73	215.3	353.3	3175.2	8665.5	0.02	0.61	0.37
185	0.3	AO-CO	125.3	2.31	2.6	2.9	188.3	45.1	67.4	133.7	5121.2	136.3	223.7	367	3254.2	8563.7	0.02	0.6	0.38
186	0.3	AO-CO	123.7	2.23	2.5	2.8	199.5	45.1	66.1	133.7	5194.2	136.3	223.7	367	3235.8	8629.6	0.02	0.6	0.38

187	0.4	AO-CO	132.3	2.02	2.3	2.5	207.6	47.8	68.7	133.7	5048.9	136.3	232.4	374.1	3481.5	8737.9	0.02	0.58	0.4
188	0.4	AO-CO	121.7	2.23	2.5	2.7	189.6	43.5	64.8	131.2	5222.9	133.73	219.4	360.1	3268.5	8681	0.02	0.6	0.38
189	0.4	AO-CO	124	2.27	2.5	2.8	179.7	44.3	66.1	133.7	5205.2	136.3	223.7	367	3243.9	8628.8	0.02	0.6	0.38
190	0.6	AO-CO	128.4	2.18	2.4	2.7	198.9	44.3	67.4	133.7	5093	136.3	228	381.3	3372.3	8664.3	0.02	0.59	0.39
191	0.6	AO-CO	136.7	2.36	2.6	2.9	192.5	46.9	71.3	138.9	4860.5	141.6	236.8	396.1	3434	8487	0.02	0.57	0.41
192	0.6	AO-CO	130.9	2.31	2.6	2.9	200.4	45.1	68.7	136.3	5025	138.92	232.4	381.3	3333.6	8559	0.02	0.59	0.39
193	0.8	AO-CO	134.8	2.54	2.9	3.2	182.7	44.3	68.7	136.3	4830.4	138.92	236.8	396.1	3365	8378.1	0.02	0.58	0.4
194	0.8	AO-CO	131.7	2.18	2.4	2.7	203.1	44.3	68.7	136.3	5013.2	138.92	232.4	388.6	3400	8616.3	0.02	0.58	0.4
195	0.8	AO-CO	130.9	2.5	2.8	3.1	188.2	42.6	68.7	136.3	4974	138.92	232.4	388.6	3291.3	8453.5	0.02	0.59	0.39
196	1	CO	141.7	3.02	3.4	3.9	156.8	45.1	71.3	138.9	4515.2	141.6	241.4	411.5	3409.5	8081.5	0.02	0.56	0.42
197	0.1	AO-RO	115.8	2.69	3	3.4	168.6	41.8	66.1	133.7	5271.6	136.3	211.2	353.3	2945	8385.2	0.02	0.63	0.35

198	0.1	AO-RO	122	2.69	3	3.3	156.5	45.1	67.4	133.7	5042	136.3	215.3	360.1	3152.2	8350.8	0.02	0.6	0.38
199	0.1	AO-RO	124.6	2.5	2.8	3.1	169.6	45.1	68.7	133.7	5082.4	136.3	219.4	367	3211.3	8463.4	0.02	0.6	0.38
200	0.2	AO-RO	124.4	2.5	2.7	3	172.5	45.1	68.7	133.7	5085.9	136.3	219.4	367	3203.3	8461.7	0.02	0.6	0.38
201	0.2	AO-RO	117.4	2.69	3	3.3	150.2	42.6	66.1	133.7	5195.1	136.3	211.2	360.1	3023.4	8368.8	0.02	0.62	0.36
202	0.2	AO-RO	123	2.54	2.9	3.1	163.7	44.3	67.4	133.7	5101.3	136.3	219.4	360.1	3148	8413.1	0.02	0.61	0.37
203	0.3	AO-RO	125.5	2.5	2.8	3.1	171.3	45.1	68.7	136.3	5107.2	138.92	223.7	367	3173.7	8452.2	0.02	0.6	0.38
204	0.3	AO-RO	119.2	2.54	2.8	3.1	171	43.5	66.1	133.7	5223.4	136.3	215.3	360.1	3097	8491.4	0.02	0.62	0.37
205	0.3	AO-RO	123.6	2.69	3	3.3	161.8	44.3	67.4	133.7	5062.3	136.3	219.4	367	3173.5	8397.6	0.02	0.6	0.38
206	0.4	AO-RO	126.4	2.54	2.8	3.1	175.4	45.1	68.7	136.3	5028.1	138.92	223.7	374.1	3207.6	8411.1	0.02	0.6	0.38
207	0.4	AO-RO	124.3	2.64	2.9	3.3	160	44.3	68.7	136.3	5032.5	138.92	219.4	374.1	3143.3	8335.8	0.02	0.6	0.38

208	0.4	AO-RO	120.4	2.8	3.1	3.5	155.7	42.6	67.4	133.7	5147.2	136.3	215.3	360.1	3067.3	8370.2	0.02	0.62	0.37
209	0.6	AO-RO	126.7	2.59	2.9	3.3	176.8	45.1	68.7	136.3	4991.6	138.92	223.7	381.3	3212.4	8380.9	0.02	0.6	0.38
210	0.6	AO-RO	119.9	2.91	3.3	3.7	147.6	42.6	67.4	136.3	5131.9	138.92	215.3	367	3008.8	8288.2	0.02	0.62	0.36
211	0.6	AO-RO	120.1	2.8	3.1	3.5	158.2	42.6	67.4	136.3	5144.5	138.92	215.3	367	3040.4	8343.1	0.02	0.62	0.36
212	0.8	AO-RO	120.6	2.69	3	3.4	177	42.6	67.4	138.9	5179.2	141.6	219.4	374.1	2999.5	8355.7	0.02	0.62	0.36
213	0.8	AO-RO	126.6	2.85	3.2	3.6	157.1	44.3	70	138.9	4972	141.6	223.7	381.3	3102.7	8231.8	0.02	0.6	0.38
214	0.8	AO-RO	121.4	2.85	3.2	3.6	160	41.8	67.4	136.3	5106.4	138.92	219.4	374.1	3009.5	8276	0.02	0.62	0.36
215	1	RO	131.1	2.96	3.3	3.7	155.1	44.3	71.3	138.9	4806.3	141.6	228	396.1	3171	8132.4	0.02	0.59	0.39
